# Omnivory in birds is a macroevolutionary sink

**DOI:** 10.1038/ncomms11250

**Published:** 2016-04-07

**Authors:** Gustavo Burin, W. Daniel Kissling, Paulo R. Guimarães, Çağan H. Şekercioğlu, Tiago B. Quental

**Affiliations:** 1 Departamento de Ecologia, Instituto de Biociências, Universidade de São Paulo, 11294, 05422-970 São Paulo, Brazil; 2 Institute for Biodiversity and Ecosystem Dynamics (IBED), University of Amsterdam, PO Box 94248, 1090 GE Amsterdam, Netherlands; 3 Department of Biology, University of Utah, Salt Lake City, Utah 84112, USA; 4 College of Sciences, Koç University, Rumelifeneri, Sariyer, 34450 Istanbul, Turkey

## Abstract

Diet is commonly assumed to affect the evolution of species, but few studies have directly tested its effect at macroevolutionary scales. Here we use Bayesian models of trait-dependent diversification and a comprehensive dietary database of all birds worldwide to assess speciation and extinction dynamics of avian dietary guilds (carnivores, frugivores, granivores, herbivores, insectivores, nectarivores, omnivores and piscivores). Our results suggest that omnivory is associated with higher extinction rates and lower speciation rates than other guilds, and that overall net diversification is negative. Trait-dependent models, dietary similarity and network analyses show that transitions into omnivory occur at higher rates than into any other guild. We suggest that omnivory acts as macroevolutionary sink, where its ephemeral nature is retrieved through transitions from other guilds rather than from omnivore speciation. We propose that these dynamics result from competition within and among dietary guilds, influenced by the deep-time availability and predictability of food resources.

## Abstract (web summary)

Diet is known to influence speciation, but much less is known about how this process operates at macroevolutionary scales. Using a global dietary database of birds, Burin *et al*. show that omnivory is associated with higher extinction and lower speciation rates compared to other guilds.

## Graphical abstract

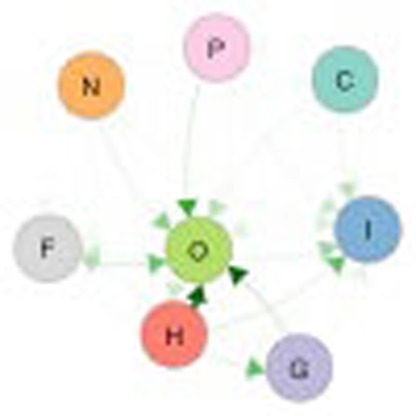

Variation of biodiversity across space and time is a trademark of the history of life on Earth and ultimately determined by speciation and extinction rates^1^,^2^. To better understand the dynamics of biodiversity we need to understand the roles of biotic and abiotic factors in determining speciation and extinction dynamics^3^. While examples of abiotic factors affecting diversification dynamics are numerous (for example, ref. 4 and references therein), few studies have explored biotic influences on macroevolutionary rates across large spatio-temporal scales^5^,^6^,^7^. Hence, the relevance of biotic interactions for diversification dynamics across deep timescales is still an open question.

Understanding the role of biotic interactions is a daunting task, given the myriad of interactions (for example, antagonistic, mutualistic and competitive) that individuals of a given species can have with individuals of other species. However, characterizing and understanding the trophic habits of species is tractable and may also be of great importance to understand potential adaptive responses to food availability, (for example, ref. 8), as well as the effects of biotic interactions on macroevolutionary dynamics^9^. As such, the diet of a given species can be used as a first-order proxy to biotic interactions. It summarizes distinct morphological, physiological and behavioural traits of an organism, which jointly determine the way it interacts with the biotic and abiotic environment^10^,^11^,^12^. For example, birds that attend army ant raids have to deal with the unpredictability of those raids, and have developed cognitive and behavioural adaptations to surpass these challenges^11^. Similarly, many nectar-feeding species have evolved beaks that suit the morphology of the flowers on which they feed, (for example, ref. 12). Since flowering phenology is strongly constrained by seasonality, the variability in climate (for example, in temperature) strongly determines geographic distributions of guilds such as nectarivores^13^. More generally, the long-term availability of particular climates^14^, as well as the spatio-temporal predictability of food resources, (for example, refs 15, 16), might influence evolutionary radiations and diversity dynamics^14^,^15^, with environmental instability setting a potential limit to the degree of specialization^17^.

Dietary strategies have been crucial for understanding species formation because interspecific competition for similar food resources can explain character displacement and the evolutionary divergence of species^18^,^19^. Nevertheless, to date only few studies at macroevolutionary scales have tested how diets might affect diversification dynamics across whole clades, (for example, refs 9, 20, 21). The paucity of whole-clade investigations relating diet to macroevolutionary dynamics is partly due to the lack of data, but also due to methodological limitations. However, recently developed methods now allow us to explicitly address^22^,^23^,^24^ or indirectly assess^25^,^26^ the relationship between trait evolution and diversification rates, and various authors have therefore analysed this relationship. Collectively, those studies revealed the effects of numerous traits on diversification dynamics, including self-incompatibility in Solanaceae^27^, tank formation and photosynthesis type in bromeliads^28^, migratory behaviour in birds^29^ and diet in mammals^9^,^20^. Hence, ecological and life history traits play a critical role for understanding macroevolutionary dynamics and broad-scale patterns of species coexistence^24^,^30^.

One of the few papers that explicitly addressed the effect of diet on macroevolutionary dynamics has shown that coarse trophic levels (that is, herbivores, carnivores and omnivores) are characterized by different diversification rates in mammals^9^. These results suggest that omnivorous mammals have lower net diversification rates than carnivores and herbivores, and that transitions into omnivory are more frequent than into other trophic levels. Using a finer diet classification within ruminants (that is, giraffes, deer, buffaloes, antelope and relatives), it has further been shown that different feeding styles underwent differential diversification rates^21^. However, this analysis suggested that grazing and mixed feeding (a combination of browsing and grazing) have both higher diversification rates and more transitions into and from these diets than browsing. Overall, these studies highlight the potential association between dietary guilds and diversification dynamics, but they also suggest that a more generalist diet (for example, omnivory or mixed feeding) might not have the same straightforward macroevolutionary outcomes at different lineages or hierarchical levels.

Birds represent a good model system for investigating the role of diet on speciation and extinction^18^,^19^, and more broadly, to understand the interplay between ecology and diversification. The clade Aves has an enormous taxonomic diversity (c. 10,300 species) with a large variability in ecological and life history traits^13^,^30^,^31^. The recently published whole-clade bird phylogeny^32^ and the abundance of ecological information for Aves have allowed biologists to assess the evolutionary dynamics (either trait-dependent or trait-independent) of many bird lineages at different taxonomic levels, (for example, refs 29, 33, 34). Moreover, different types of diet have evolved multiple times within the clade^13^. Dietary adaptations range from specialized feeders such as some insectivores (for example, swifts and swallows), frugivores (for example, oilbirds), seed predators (for example, macaws), vertebrates (for example, peregrine falcons) and carrion-feeders (for example, vultures) that feed preferentially on one particular food type to omnivores such as the medium-sized, common raven *Corvus corax* (family Corvidae), which have a generalized diet by feeding on multiple food items such as insects, fruits, seeds, vertebrates and carrion^13^. Such variation in the degree of diet specialization is probably related to different physiological and anatomical adaptations required to deal with different food items^11^,^12^,^35^. For example, some nectarivorous and frugivorous species show specific preferences for different sugar contents related to enzyme activity and absorption rates^36^, which might eventually affect their food preference and hence their degree of specificity.

Here we combine the most complete bird phylogeny^32^ and a comprehensive global data set of the diets of the world's bird species (ref. 31, updated with ref. 37) to investigate the potential effect of different diets on the speciation and extinction rates of birds, and the evolutionary transition rates between all dietary guilds. Given that shifts to new diets result in different ways of interacting with the environment^8^,^38^, and that such shifts might also affect the degree of specialization within a given lineage^9^,^21^,^39^, we hypothesize that the evolution of different diets in birds will result in distinct speciation, extinction and transition dynamics. Even though a simple classification of diet has been shown to affect diversification rates of mammals, (for example, ref. 9), we know virtually nothing about the macroevolutionary effects of diet on such diverse groups of vertebrates such as birds where we have a more refined dietary categorization. Hence, investigating the role of diet on bird diversification will not only allow us to understand its effect on this extremely diverse lineage but also help us to begin evaluating how general the observed effects of diets are for macroevolutionary dynamics across tetrapods.

For our analyses, we assigned each species to a different dietary guild based on its main diet (at least 50% of one particular food type; see also Supplementary Fig. 10 and Supplementary Note 1) for a sensitivity analysis regarding this dietary classification. When no item comprised more than 50% of the whole diet or if a given species consumed two food types equally, then it was considered an omnivore. By following this categorization we grouped species into carnivores, frugivores, granivores, herbivores, insectivores, nectarivores, omnivores, piscivores and scavengers. We then fitted Multiple State Speciation and Extinction (MuSSE) models in a Bayesian framework for 200 randomly sampled phylogenetic trees to incorporate phylogenetic uncertainty, and used the posterior distributions of diversification and transition rates to infer the relationship between diet and diversification (see also Supplementary Material for model testing, adequacy tests and sub-clade analysis). In addition, we used network analysis to further quantify the evolutionary diet transitions among guilds and a principal component analysis (PCA) of diet scores to assess the multidimensional similarity of diets. Our results indicate that dietary habits have influenced the diversification dynamics of birds, with omnivores experiencing higher extinction, lower speciation, and with transition rates being substantially higher into omnivores than into any other guild.

## Results

### Dietary guilds

Bird species are not equally distributed among dietary guilds. Both the total number of species and the phylogenetic signal strength differs among guilds (Table 1). This suggests that different dietary guilds might in fact have different diversification dynamics. The three most common dietary guilds are insectivores (55%), omnivores (12%) and frugivores (12%), and the least common is the scavenger guild (0.3%). Below we exclude scavengers from the results and discussion because their diversification rates were poorly estimated due to small sample size (33 species grouped in a few lineages such as New World and Old World vultures, some crows and a few phylogenetically isolated species). In general, all dietary habits seem to have multiple origins in Aves. However, there are at least two distinct evolutionary conservatism patterns in diets across the bird tree of life. Whereas omnivores are largely spread randomly across the bird phylogeny, all other dietary guilds are phylogenetically clustered to some extent (Table 1 and Supplementary Figs 1–9).

### Diversification rates

**Figure 1:**
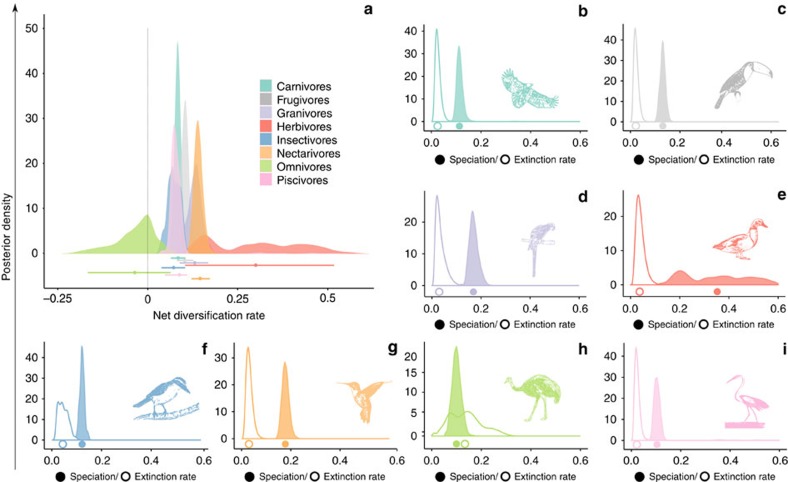
Diversification rates associated with different bird dietary guilds. (**a**) Posterior distributions of net diversification rates for each dietary guild and (**b**–**i**) corresponding posterior distributions of speciation and extinction rates. Bars on **a** represent the 95% CI of each distribution and the dots the median of the posterior distribution. In **b**–**i** colour-filled curves represent speciation rates and white-filled curves represent extinction rates. The filled and empty dots represent median values for speciation and extinction rates, respectively.

Our results reveal that the net diversification rate of omnivores is lower than that of any other dietary guild (Fig. 1a). Underlying these dynamics is a lower speciation rate and a higher extinction rate of omnivores compared with other guilds (Fig. 1b-i). In addition, the net diversification rates for all dietary guilds are positive except for omnivores, where the median value of the net diversification rate is negative (Fig. 1a). Even though the distribution of net diversification rates for omnivores includes zero (specifically when looking at the posterior distribution peak, Fig. 1a), this guild is the only one that has a large portion of negative values in its diversification rate posterior distribution. This reinforces the idea that omnivores have different dynamics, with net diversification rates being significantly lower than in other guilds.

The posterior distributions of all rates for almost all guilds are mono-modal, suggesting that parameter values well represent the estimated value for each rate. The main exception is the speciation rate for herbivores (Fig. 1e). Other distributions that are not mono-modal are the extinction rates for both insectivores and omnivores (Fig. 1f-1h respectively). In the case of herbivores, the distribution has a large uncertainty that results from combining mono-modal posterior distributions for individual phylogenetic trees that converged into different values. For insectivores and omnivores, the bimodality of the extinction posterior distributions also arises from combining several mono-modal distributions from all sampled trees. However, this bimodality represents the effect of phylogenetic uncertainty and not the non-convergence of estimates, reinforcing the importance of our implemented modelling framework, which explicitly includes sources of phylogenetic uncertainty.

**Figure 2:**
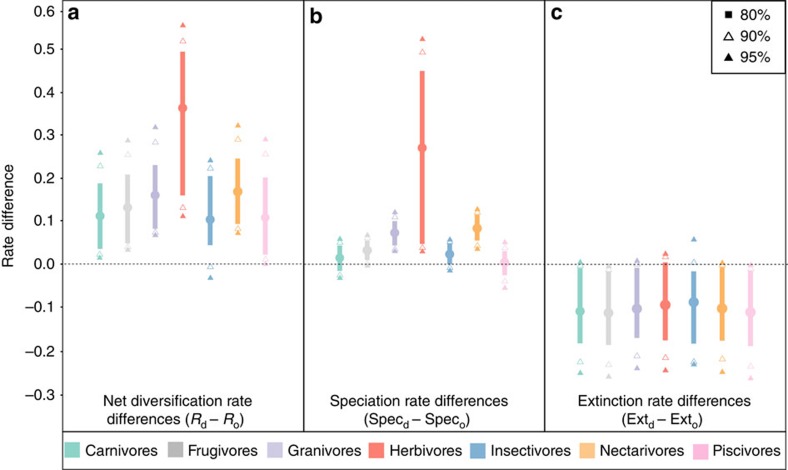
Distinct diversification dynamics associated with omnivory in birds. Differences between net diversification (**a**), speciation (**b**) and extinction (**c**) of all dietary guilds relative to omnivores. The differences in rates are calculated by incorporating phylogenetic uncertainty and therefore represent a posterior distribution of differences between the rate estimates of each guild compared with the rate estimate of omnivores. The thick lines, filled and hollow triangles indicate different credibility intervals, and the dashed line indicates 0 (no difference). Positive values mean that the considered rate is higher for each guild than the same rate for omnivores. Omnivores generally show lower speciation and higher extinction rates, although differences are significant at different degrees of credibility depending on the guild.

Figure 2 shows the credibility intervals (CIs) at different significance levels (95, 90 and 80%) for the posterior distributions of differences between the rates of all guilds as compared with those of omnivores. This reveals that net diversification, speciation and extinction of omnivores differ from other guilds in most cases (Fig. 2). Omnivores show a statistically significant lower diversification rate than all other guilds except insectivores where this difference is marginal (Fig. 2a). A similar pattern is found in speciation rates, where omnivore rates are lower than those of granivores, herbivores, nectarivores and frugivores (at 95% CI for the first three guilds and 90% CI only for the latter; Fig. 2b). Even though extinction rate differences are not as striking for some guilds as those for speciation rates (compare Fig. 2c and Fig. 2b), omnivores show higher extinction rates than carnivores, frugivores, granivores, nectarivores and piscivores (at a 90% CI). Omnivores also have extinction rates that are marginally higher than those of herbivores and insectivores.

**Figure 3:**
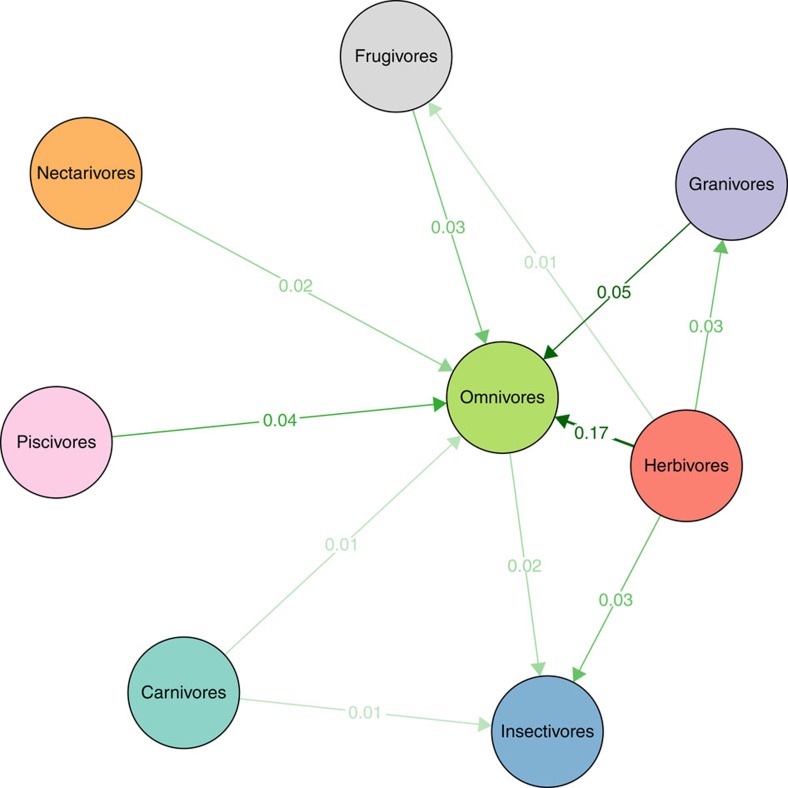
Transition rates among different bird dietary guilds. Network depicting the estimated transition rates (links) between dietary guilds (nodes). The intensity of each directed link is proportional to the median of the posterior distributions of transition rates. All transition rates smaller than 0.001 were omitted in the figure for better visualization. Numbers above the links correspond to the median value of the posterior distribution of the corresponding rate. Transitions towards omnivores are more common than any other direction of transition, and omnivory is the only guild that is significantly more connected than expected by chance (null-model analysis, *P*<0.0001).

Quantifying the transitions into different dietary guilds reveals a prevalence of transition rates into omnivores rather than into any other dietary guild (Fig. 3 and Supplementary Figs 11 and 12). Herbivores and granivores show the highest transition rates into omnivory, insectivores almost no transitions into omnivory or any other diet, and other dietary guilds intermediate transition levels into omnivory. Overall, these results suggest that all dietary guilds preferentially shift into omnivores, except insectivores. This is also supported by a network analysis that shows that eigenvector centrality (a measure of whether network nodes—here dietary guilds—behave as preferential end points within a network) of omnivores is equal to 1, which is significantly higher than expected by chance (permutation test with 10,000 permutations, *P*<0.0001; Supplementary Fig. 10). Estimates for all other guilds show centrality values that are not significantly different from the null model (Supplementary Fig. 10). It is interesting to note that the estimates of transition rates into omnivory suggest that the overall rate of transition into omnivory (summing up the transitions from all guilds) is at the same order of magnitude as the speciation rates for other guilds (compare the panels b–i of Fig. 1 with Fig. 3).

### Dietary niche overlap

Each species has its dietary preferences described by a vector of diet items (that is, vertebrates, fruits, seeds, invertebrates and so on) whose scores sum up to 10, and each of these scores represent the proportion that a given food item is consumed in the diet of a given species. To explore the multidimensional dietary similarity among guilds we used a PCA on the complete vector of diets for each species. This analysis shows that within the first three PCA axes the omnivores occupy intermediate positions relative to all other dietary guilds, having a considerable overlap with them. In contrast, other guilds show little overlap with each other at least in one of the three PCAs (Supplementary Fig. 14). Higher overlap of omnivores with other guilds is also reflected in the mean Euclidean distance between each species in the orthogonal space formed by the first three PCA axes (Table 2). Omnivorous species show greater mean distance within their own guild than do non-omnivorous species within their own guilds. In addition, average distances between omnivores and species within each other guild are usually similar while the average distances between species of specialized guilds and of other guilds (including omnivores) can be highly variable and for many comparisons higher (Table 2). These results mean that omnivore guild is more centrally positioned in a coarse dietary space (Supplementary Fig. 14). Finally, we also explored the patterns of overlap between omnivores and species of other guilds. All omnivores include at least some insects in their diet (Supplementary Fig. 15). Fruits and grains also show considerable prevalence in their diet, but carrion is rarely consumed by omnivores (Supplementary Fig. 15). Overall, these results support the idea that diet overlap of omnivores with other guilds is high.

### Model performance and adequacy

Our four auxiliary analyses showed that, in our particular case, it is very unlikely that the statistical methods (MuSSE) and the diet classification scheme produced spurious associations between diet and diversification dynamics. First, we show that simulations using empirical transition rates and no association between speciation/extinction rates and trait states do not recover the speciation and extinction dynamics seen in the empirical analyses (Supplementary Figs 16 and 17). Second, a model adequacy test suggested that the simulations using all estimated parameters produced a range of diet proportions that encompass the proportions of diets as observed in the empirical data set (Supplementary Fig. 18). Third, the diversification dynamics observed in sub-clades of the whole phylogenetic tree showed partial concordance with our main results, especially that extinction rates of omnivores tend to be higher than those of any other dietary guild (Supplementary Fig. 19). The results for speciation and transition rates within species-rich sub-clades (Passeriformes, Piciformes, Psittaciformes and Charadriiformes) were inconclusive and difficult to interpret (Supplementary Results and Supplementary Fig. 19, and Supplementary Note 1 for a brief discussion). Higher transition rates into omnivory were sometimes also recovered in these sub-clades, but for the sub-clade analysis, as opposed to the whole-tree analysis, speciation rate became relatively more important on generating omnivore species than the transition rates. This change in relative importance (speciation being the main process of formation of new omnivore species) suggests that the speciation and transition dynamics are interrelated, making a comparison with the full phylogenetic tree not straightforward (see Supplementary Results for further discussion). Finally, we performed a fourth test to evaluate the sensitivity of our diet classification scheme. Using a more inclusive categorization of omnivory did not change our main results, that is, that omnivory can be seen as a macroevolutionary sink (Supplementary Figs 20–24). Hence, for the analysis presented here we suggest that the MuSSE model provides reliable rate estimates and that the qualitative results and conclusions derived from the whole-tree analysis are robust. We therefore focus the discussion only on the main results.

## Discussion

Diet has a clear association with the diversification dynamics of birds. Most prominently, omnivores show lower (and even negative) net diversification rates compared with the positive rates of all other guilds (Fig. 1a). Our results suggest that this distinct evolutionary dynamic exhibited by omnivores arises from the interplay between significantly lower speciation rates and significantly higher extinction rates when compared with other guilds (Fig. 2). Estimating speciation and extinction rates from molecular phylogenies has limitations (ref. 40, but see ref. 41), but we highlight that our main conclusions are based on qualitative differences between omnivores and other dietary guilds rather than on the precise rate estimates. Interestingly, we further observed that transitions into omnivory occur at much higher rates than into any other guild (Fig. 3) and that those transition rates occur in the same order of magnitude as the estimated speciation rates of other guilds. This result suggests that omnivory acts as a macroevolutionary sink where generalized diets are only transient. This sink behaviour might be a more widespread pattern in tetrapods because similar dynamics have also been suggested for mammals^9^.

Lower speciation rates and higher extinction rates of omnivores in mammals^9^ were obtained by defining omnivory as eating similar proportions of plant and meat compared with two other trophic levels (that is, carnivores and herbivores). However, at lower taxonomic levels within the mammalian tree of life such results differ among lineages. For instance, diversification rates have also been found to be lower for more generalized bat lineages that complement their frugivorous diet with other food items (that is, nectar and pollen) relative to more specialized frugivore lineages^20^. In contrast, in ruminants the grazing and mixed-feeding strategies have both higher diversification rates than browsing^21^. In general, lower diversification rates of omnivores could be explained by the ecological tenet that generalist species might be at a disadvantage when competing with specialists^42^. Such a ‘jack of all trades is a master of none' mechanism (species that can utilize several resources while performing poorly at utilizing specific resources^43^) could leave a signature at the macroevolutionary scale.

According to ref. 44, two main characteristics determine the coexistence probability of two or more species in the same place: niche overlap and competition asymmetry. We suggest that omnivorous species are at competitive disadvantage relative to species of more specialized guilds due to both factors. For niche overlap, our diet similarity analysis shows that omnivorous birds have a considerable degree of diet overlap with species from at least two other dietary guilds. In fact, omnivorous species have, on average, equivalent distances to other omnivorous species or to species belonging to other guilds (Table 2), indicated by similar average pairwise distances. In addition, omnivory has a more central position than other guilds on all three PCA axes of the diet analysis, and always some degree of overlap with other dietary guilds (Supplementary Fig. 14). When considering competitive asymmetry, species within specialized dietary guilds should also show different levels of specialization. For example, within insectivores there are some highly specialized lineages. True antbirds (Thamnophilidae) are specialized on eating mostly terrestrial invertebrates escaping from army ant raids in tropical forests^11^, and flycatchers (for example, family Tyrannidae) are highly adapted to catching their insect prey in flight. Dietary specialization therefore plays an important role for competitive dynamics and thereby might also influence evolutionary dynamics.

From an ecological point of view, several authors have proposed that the fitness of specialists (usually assessed via population size) is higher when compared with generalists^45^,^46^. This can be explained by trade-offs between performing well at acquiring a narrow range of resources (for example, hosts, food items and so on) or having a wide range of resources at the cost of being worse at acquiring them. When a specialist and a generalist species compete for the specialist's preferred resource, the specialist species should ecologically outperform the other^46^. This explanation might be particularly true if resources are constantly available, for example, in relatively stable or aseasonal environments. In contrast, specialists might be at disadvantage in places or at times where the preferred resource is scarce or unpredictable^18^,^45^.

If we expand this competitive scenario to a situation where omnivores share their resources with multiple different specialists, we hypothesize that over longer timescales omnivores would be systematically at a competitive disadvantage due to both high niche overlap and competition asymmetry. This would ultimately lead to very low abundances of generalist species^46^ and possibly to local extinctions^47^. The simultaneous competition with multiple species might therefore translate into higher extinction rates at a macroevolutionary scale, resulting in a high macroevolutionary cost to omnivores. Assuming this scenario of multi-species competition, an omnivore would be a ‘jack of all trades' (a species that can utilize several resources^43^) trapped in an arena of ecological competition with multiple competitors belonging to different guilds. Such a ‘master of none' mechanism (that is, species perform poorly at utilizing specific resources^43^) would lead to macroevolutionary consequences at the species level, where the ‘jacks of all trades' should show low speciation and/or high extinction rates.

As outlined above, we suggest that higher extinction rates of omnivorous birds are the result of competition with species from multiple guilds. However, the generality of such a mechanism remains to be tested more widely given that a potential association between lower diversification rate and a more generalized dietary guild has so far only been examined in mammals, (for example, ref. 9) and birds. Assuming that body size is a proxy for ecological niche^48^, mammals might be responding to a similar mechanism as proposed here. Mammalian omnivorous species show both lower diversification rates^9^, as well as intermediate and overlapping body masses (that is, intermediate and overlapping ecological niches) when compared with herbivores and carnivores^49^. Hence, inter-guild competition might be an overlooked mechanism that is potentially important to explain lower diversification rates of omnivorous species. Although species-level mechanisms or outcomes were at first widely rejected as being drivers of macroevolutionary dynamics, they are now considered important mechanisms^6^,^50^,^51^ and the ever-growing empirical studies that show a pattern of trait-based diversification^21^,^27^,^29^ suggest that it might indeed be a common phenomenon in determining the evolutionary success of lineages with different traits^2^,^51^.

Along with increased extinction rates, we also detected lower speciation rates of omnivores relative to other guilds. The mechanism behind the association between low speciation rates and omnivory is more elusive, but given that speciation and extinction rates are usually linked by the same mechanisms^52^,^53^, it is possible that inter-guild competition might also play a role here. If each guild is an adaptive zone (*sensu* (ref. 54)), where the speciation process results in the crowding of this adaptive zone, then higher rates of speciation from multiple specialized guilds might result in a compound ‘crowding' effect that reduces speciation rates of omnivores at the macroevolutionary scale. Alternatively, the lower speciation rates of omnivores could also be explained by higher extinction rates at the population level, whereby populations experiencing high competition with multiple species are likely to go extinct. In this scenario, some populations that are going through a speciation process might not have enough time to be fully separated into two different species, resulting in lower speciation rates at the macroevolutonary scale, a process that might be referred to as ‘ephemeral speciation'^55^.

Given the macroevolutionary ‘costs' associated with omnivory (that is, low speciation rates and high extinction rates), it might seem surprising that this dietary guild still constitutes such a considerable portion of extant bird diversity (1,158 species, circa 12%; Table 1). From a deep-time perspective, a lineage with low diversification rates—especially those with negative rates—should eventually disappear or at best reduce its diversity due to species sorting^6^,^56^. We hypothesize the reason why omnivory has not disappeared lies in the high transition rates into omnivory.

Our results show that transition rates into omnivory are significantly higher than into any other dietary guild (Fig. 3) and that they occur at the same order of magnitude as the speciation rates for other guilds. Moreover, network analysis reveals that omnivory is the most central guild and that diet shifts occur from all other dietary guilds into omnivory more than one would expect by random (Supplementary Fig. 10). This suggests that omnivore lineages preferentially originate at the macroevolutionary scale via transitions, and not through speciation. The reason why such transition rates are so high could depend on selection driven by resource competition at the individual level. Omnivory could be favoured at times or places with low abundance of a preferred resource or when resource availability is highly unpredictable^17^,^45^. For instance, climate variability (for example, seasonality) clearly influences resource availability, and specialists might only survive if their resources are continuously available and highly predictable^15^,^17^. For example, specialized nectarivores and frugivores only survive in places where seasonality is low and hence resource availability relatively constant^13^. Granivores benefit in dry climates where seeds are constantly available, whereas insectivores perform well in the tropics where insects are available all year round^13^. Hence, a low spatio-temporal predictability of resources, as well as high environmental instability is likely to benefit omnivores by setting a limit to the degree of specialization^17^. At macroevolutionary scales, this will influence diversification dynamics and increase transition rates into omnivory.

A mixed-feeding diet has been shown to be beneficial for individuals belonging to different herbivore species across different animal groups^57^,^58^,^59^. If individual-level selection is indeed an important factor for avian transitions into omnivory, we can expect ancestral lineages to feed on resources that were temporally limited, unpredictable, difficult to digest or with poor nutrition. In birds, most transitions into omnivory come from granivores and herbivores, and herbivores are represented with only few species (Table 1). Given such low frequency and the fact that feeding exclusively on leaves might represent a poor diet^60^, selection pressure to add new, perhaps more nutritious, food items could indeed drive the evolution of omnivory from herbivorous ancestral lineages. In the case of granivores, it is more likely that resource availability plays an important role, but analogously to the hypothesis of transitions from herbivores this hypothesis remains to be properly tested. Interestingly, the transitions into omnivores (Fig. 3) and the detailed information on their diets (Supplementary Fig. 15) suggest that transitions into omnivory systematically include the addition of insects. Insects might represent a predictable and protein-rich resource, but insectivory might also pose evolutionary challenges such as the digestion of lipids^61^ and the potential competition with more specialized insectivore species^11^.

We propose that the diversification dynamics of different dietary guilds are driven by resource competition caused by deep-time temporal and spatial changes in resource availability and predictability. These fluctuations in resource availability and predictability might create evolutionary pressures at two levels of organization. At the individual/population level, these fluctuations might promote transitions into omnivory in times of food resource scarcity by selecting individuals/populations that do not rely on single food items^62^. At the species level, the same climate and resource fluctuations might result in more favourable conditions that would eventually bring back omnivore species in contact with species belonging to multiple dietary guilds. In times or places with relatively small changes in resource availability and predictability, the more specialized guilds can rapidly (re)colonize areas where omnivores emerged, possibly preventing the transitions of omnivores back into other more specialized guilds due to the velocity of migration in relation to selection. This would explain the higher extinction rates of omnivores. Such a selection mosaic (*sensu* (ref. 63)) of resource distribution and competition would therefore mediate the macroevolutionary fate of omnivores and specialized dietary guilds^9^.

Even though it is challenging to directly test mechanistic hypotheses at a macroevolutionary scale, we suspect that such a competitive mechanism acting at both the species and individual level should not only result in specific macroevolutionary patterns (for example, higher extinction rates of omnivores) but also in macroecological predictions. At broad spatial scales, we therefore predict that the spatial distribution of omnivorous species peaks in places where co-occurrence of specialized dietary guilds is low. For instance, the relatively stable, long-term (Cenozoic) availability of rainforest climates in South America^14^ coincides with a low diversity of omnivores and high diversity of species belonging to specialized dietary guilds such as granivores, frugivores, nectarivores, insectivores and carnivores^13^.

Expanding these ideas into the Anthropocene where human-driven global change is homogenizing biological communities and eliminating the resources of many specialist species, we expect that a shift in the competitive dynamics between generalists and specialist species will occur. Globally, generalist bird species are at a much lower risk of extinction than specialists, and in birds there is a positive relationship between increased specialization and increased risk of human-driven extinction^64^. Hence, ongoing human-driven changes are likely to distort future macroevolutionary dynamics by changing diversification rates and favouring generalist species at the expense of specialists.

Irrespective of the mechanism, our results support the notion that omnivory is a macroevolutionary sink, that is, a transient state in bird evolutionary history. This dynamic seems to be affected by two different hierarchical processes. On the one hand, species sorting through higher extinction rates and lower speciation rates will lower species richness of omnivores through time. On the other hand, selection — presumably driven by changes in resource abundance and predictability — brings species diversity of omnivores back and results in higher transition rates into omnivory at the macroevolutionary scale. The ecological mechanisms behind these macroevolutionary dynamics are difficult to test, but the available data suggest that the interplay between intra- and inter-guild competition might lie at the heart of this macroevolutionary game of the ‘jack of all trades is a master of none'.

## Methods

### Data set

We used the bird phylogeny from Jetz *et al*.^32^, which encompasses almost all bird species (9,993 species, available online at http://birdtree.org/). The tree was built using molecular data from 6,670 species, and the remaining taxa with no molecular information were added to the phylogeny based on taxonomic information and simulated branching times from a pure birth (Yule) model of diversification^32^. The distribution of these inserted species spans the entire tree and virtually all clades (Supplementary Fig. 13). The addition of those species should therefore not bias diversification estimates and at best only homogenize any real differences between different traits, making our tests more conservative with respect to finding true differences in diversification dynamics among guilds. A distribution of 10,000 trees with different topologies was obtained from the original paper^32^. To account for phylogenetic uncertainty, we randomly sampled 100 trees from this posterior distribution of trees for each of the two backbone trees, totalling 200 trees. Using these 200 trees diminishes any possible biases that the insertion process of species with no molecular data could bring into the phylogeny. We note that the two backbones from ref. 32 showed a similar amount of differences in topology as when both were compared with two other recently published high-order bird phylogenies (Supplementary Fig. 25 and Supplementary Notes for methods, see Supplementary Methods).

A comprehensive bird diet database (ref. 31; updated with ref. 37) was used with numerical scores for different food types consumed by birds (including invertebrates, fruits, nectar, seeds, terrestrial vertebrates, fishes, carrion, plants (non-reproductive) and miscellaneous). The data came from over 250 ornithological books, as well as peer-reviewed articles compiled in a global ornithological database by C.H.S. (ref. 31; updated with ref. 37). The literature used includes synthetic works (for example, *Handbook of the Birds of the World*, *The Birds of Africa*, *The Birds of South America*, *Australia/New Zealand Handbook of Birds*, *The Birds of Western Palearctic* and all the books on bird families), which provide bird species accounts based on a summary of all literature on a particular bird species. Therefore, our diet classification was based on a comprehensive diet database that summarizes dietary preferences across a species range and across seasons. The scores of all diet items add up to 10 and represent the approximate proportion of each food type in the diet of a given species. A species was classified into a specific dietary guild if it had one food item with a score >5 (for sensitivity analysis see Supplementary Figs 19–23). Species with only two equally consumed food items in their diet or with no food item with a score >5 were classified as omnivores. Thus, all species were classified into nine dietary guilds: carnivores (feeding predominantly on vertebrates); frugivores (feeding predominantly on fleshy fruits); granivores (feeding predominantly on seeds); herbivores (feeding predominantly on non-reproductive plant material such as leaves, roots and shoots); insectivores (feeding predominantly on insects or other invertebrates); nectarivores (feeding predominantly on nectar); piscivores (feeding predominantly on fish); scavengers (feeding predominantly on carrion); and omnivores (the species that do not have a predominant diet). After matching the taxonomy of species with dietary data with the phylogeny, we finally used a total of 9,876 species in all analyses.

### Model fitting and parameter estimates

MuSSE models were fitted across all sampled trees^23^. This class of models estimates the parameter values (speciation, extinction and transition rates) associated with each trait state in a phylogeny. The models were implemented in a Bayesian Monte Carlo Markov Chain (MCMC) framework to account for both phylogenetic and rate value uncertainties. Phylogenetic methods might underestimate extinction rates^40^,^65^, and to avoid rates to be equal to 0 (especially transition rates that are prone to be very small in a multi-state model) we used three Cauchy distributions as hyperpriors. These hyperpriors have a location parameter fixed to 0 and the scale parameter is estimated from MCMC analysis. This allowed rates to be very small, but not zero. All parameters were independently estimated, that is, with no constrains. A total of 1,500,000 steps (sampling every 1,000th step) were necessary to achieve an acceptable convergence of the majority of the parameters. The Bayesian analysis was run for separate trees in parallel on four computer servers. All analyses were conducted within the statistical environment R^66^ using the *diversitree* package^23^ and a new script designed to implement the MCMC analysis (available at https://github.com/dsilvestro/mcmc-diversitree).

There has been recently a debate over the performance of trait-based models^67^,^68^. The main critiques are related to the low presence of true replicas. Strong phylogenetic signal and few events of state change in a given character could lead to pseudoreplication^67^, and a high percentage of false positives in a class of trait-dependent speciation and extinction models due to rate heterogeneity throughout the tree could additionally bias rate estimates^68^. Although the latter limitation has only been proven to be true for binary-state characters, some authors suggest that it is a common limitation among all xxSSE models^67^,^68^.

As an alternative to the trait-dependent methods, a recent study by Huang and Rabosky^33^ estimated speciation and extinction rates using BAMM (Bayesian Analysis of Macroevolutionary Mixtures^26^), a trait-independent method that estimates these rates using reversible-jump MCMC to identify shifts in diversification rates. With the BAMM results a significant relationship between the degree of sexual dichromatism in birds and diversification rates was found using comparative methods. However, using BAMM as an alternative solution to xxSSE models does not seem to be fully adequate for our analyses and the phylogenetic structure of the diet traits. BAMM does not estimate transitions between states of the analysed character when estimating speciation and extinction rates, and these rates seem to have an important role for the macroevolutionary dynamics in our analyses, and more broadly in evolutionary dynamics. In addition, given the phylogenetic overdispersion of omnivory in our phylogenetic trees (omnivore species usually appear as a isolated tip within a clade with species that belong to a more specialized dietary guild) and how BAMM operates (it finds a node where a shift in rate is justifiable) we suspect that it is virtually impossible to detect rate shifts associated with omnivory using BAMM. The reason is that within each group of species that contains omnivores the statistical power to detect any shifts in speciation and/or extinction rates for omnivorous species would be insufficient. We therefore suspect that in such a phylogenetic trait configuration the diversification rates obtained with BAMM for omnivores would potentially be biased in different directions depending on the diet of closely related species. This would turn any posterior analysis unprofitable. Last, a semi-parametric test to detect trait-dependent diversification was proposed by Rabosky and Huang^69^ that relies on the rate estimates derived by BAMM to later estimate the relationship between a binary (or continuous) trait and the diversification rates. This test uses rate regime permutations to build null distributions of correlation coefficients. Even though this seems as an interesting alternative, this test was not used here since it is currently not available for multi-state discrete characters (ref. 69, page 12).

To assess the reliability of our MuSSE results in relation to the issues raised by ref. 68 we performed four additional analyses. In the first additional analysis, we tested if rate heterogeneity captured by our empirical phylogenetic trees might have led MuSSE to detect spurious relationships between trait states and diversification. To do this, we simulated the evolution of a discrete character with the same number of states as in our empirical data set on 10 randomly selected empirical bird trees, using the empirical transition rate estimates. We then tested for a statistical association between those neutral characters and the estimated rates (Supplementary Methods section A1) to see whether the model detects similar associations between trait states and speciation and extinction rates.

In the second set of additional analyses (model adequacy), we simulated 1,000 trees using the rates estimated in our main empirical analysis, to check whether the estimated empirical rate values would generate a proportion of trait (diet) states comparable to the empirical proportions (Supplementary Methods section A2 and Supplementary table 1). In the third set of additional analysis (sub-clade analysis), we ran separate MuSSE analyses for the four major bird orders (Passeriformes, Piciformes, Psittaciformes and Charadriiformes) on 10 trees to investigate if the macroevolutionary patterns associated with different diets as obtained from the whole-tree analysis were also recovered at these sub-clades (Supplementary Methods section A3). In the fourth additional analysis, we investigated the extent to which our results were affected by our dietary classification scheme. We used a different classification scheme to categorize species into discrete dietary guilds and then estimated all diversification rates using the same procedure as in our main analysis using the 10 sampled trees (Supplementary Methods section B). The complete description and results of these tests can be found in the Supplementary Methods.

### Posterior distributions of rates

The posterior distributions of parameters from all 200 trees were combined into one single posterior distribution for every parameter (for example, speciation, extinction and transition rates and hyperprior parameters, adding up to 93 parameters in total). For net diversification rates *r* (speciation−extinction), the posterior distribution was built by calculating *r* for each sample of the MCMC, resulting in the same 1,500 values for each state of the trait. For all posterior distributions of speciation, extinction and net diversification rates the 95, 90 and 80% CIs (highest posterior density) were calculated. All results and discussion do not encompass rates from scavenger species because estimates were poor due to small sample size (33 species).

### Comparison of rates of dietary guilds

To test whether or not speciation, extinction and net diversification rates of omnivores were significantly different from rates of all other guilds, we calculated the difference between each omnivore rate to the rate estimated for each other dietary guild. These differences in speciation, extinction and net diversification rates were calculated at each sampled MCMC step, building posterior distributions of differences. These distributions were then compared and analysed separately and the omnivore rate was considered different when the value 0 was not included in the CI for each rate difference comparison (Fig. 2).

The MuSSE analysis also allowed us to generate estimates for pairs of transition rates but not to explicitly test for any general asymmetry while considering all the transitions at the same time. Depending on how transition rates are organized among distinct dietary guilds, some guilds might constitute preferential routes of transition. In contrast, if there is no consistent pattern in the distribution of transition rates among guilds, no guild will show a higher transition rate into or from it. To evaluate if the empirical transition matrix significantly deviates from a null model where all transitions are expected to be balanced among nodes, we used a network theory approach. We depicted the transition rates as weighted links and dietary guilds as nodes of the transition network. If species from other guilds consistently shift to the same dietary guild, this latter dietary guild would show high levels of centrality in the transition network. In the transition network, eigenvector centrality describes how the transition rates lead, directly or indirectly, to a given dietary guild. We computed the eigenvector centrality of each dietary guild^70^, which varies from 0 (peripheral dietary guild) to 1 (central dietary guild). Thus, a highly central dietary guild can be viewed as an absorbing state to which species from other dietary guilds may evolve by changing resource use. To verify the significance of these centrality values, we built a null distribution of centrality values by randomly assigning to each link a value sampled from the estimated transition rates without replacement for each of the 10,000 replicas. We then compared the empirical centrality values to this null distribution and verified to which quantile the real value corresponded.

### Diet similarity analysis

With the original diet scores for all species, we quantified the score frequencies of each food item within the diet of all omnivore species (Supplementary Fig. 15). This was done to better characterize the diet of omnivorous species and to trace diet similarities between omnivores and all other guilds. We additionally performed a PCA using the full vector of diet scores (with each food item as a variable) to characterize omnivorous species and their multidimensional dietary similarity with other guilds (Supplementary Fig. 14). This allowed us to assess the distribution of dietary guilds in niche space with reduced multidimensionality. We also calculated the Euclidean distance between each possible pair of species in the orthogonal space created by the first three principal components (Table 2). These distances were then averaged within and between each and all guilds for further comparison.

## Additional information

**How to cite this article:** Burin, G. *et al*. Omnivory in birds is a macroevolutionary sink. *Nat. Commun.* 7:11250 doi: 10.1038/ncomms11250 (2016).

## Supplementary Material

Supplementary Information Supplementary Figures 1-25, Supplementary Table 1, Supplementary Notes and Supplementary References.

## Figures and Tables

**Table 1:** Number and percentage of total species per dietary guild and mean phylogenetic signal of each dietary guild.

Dietary guild	Number of species (% of total)	Phylogenetic signal (*D* value)
Carnivores	280 (3%)	−0.2762*
Frugivores	1,141 (12%)	−0.03879*
Granivores	824 (8%)	−0.02475*
Herbivores	189 (2%)	0.04889*
Insectivores	5,409 (55%)	−0.05087*
Nectarivores	542 (5.7%)	−0.3661*
Omnivores	1,159 (12%)	0.52702†
Piscivores	233 (2%)	−0.13848*

Phylogenetic signal (measured as character dispersion *D* of a binary trait) was averaged over 10 random trees (5 from each backbone). Negative *D* values indicate phylogenetic clustering, whereas highly positive values indicate phylogenetic overdispersion. Values not different from 0 indicate that the character evolves according to a Brownian motion process, whereas *D* not significantly different from 1 indicates randomly distributed states on the tree.

^*^Values different from 1 but not from 0.

^†^Values different from both 0 and 1; significance was the same for all trees.

**Table 2:** Average Euclidian distances between species calculated using the first three axes of a PCA on diet item scores per species within guilds (bold) and between guilds.

	Carnivore	Frugivore	Granivore	Herbivore	Insectivore	Nectarivore	Piscivore	Omnivore
Carnivore	1.296 (0.017)							
Frugivore	9.286 (0.008)	2.375 (0.004)						
Granivore	9.335 (0.009)	11.503 (0.004)	2.455 (0.005)					
Herbivore	3.723 (0.020)	7.980 (0.009)	6.770 (0.011)	2.131 (0.023)				
Insectivore	9.134 (0.004)	11.153 (0.002)	11.749 (0.002)	8.107 (0.004)	1.782 (0.001)			
Nectarivore	2.601 (0.011)	10.469 (0.005)	11.071 (0.006)	5.381 (0.013)	9.588 (0.002)	1.575 (0.008)		
Piscivore	2.261 (0.017)	8.405 (0.008)	8.408 (0.010)	2.613 (0.020)	7.695 (0.004)	3.707 (0.012)	1.368 (0.018)	
Omnivore	5.760 (0.008)	7.470 (0.004)	7.678 (0.004)	4.133 (0.009)	7.019 (0.002)	7.052 (0.005)	4.435 (0.008)	4.337 (0.004)

Omnivores show higher within-guild average Euclidian distances than other guilds, suggesting that the diet similarity observed among omnivorous species is on average lower than diet similarity seen within any other guild. Numbers within parenthesis indicate s.e.'s.
