# Supplementary material for: Omnivory in birds is a macroevolutionary sink
Source: Nat Commun. 2016 Apr 7;7:11250. doi: 10.1038/ncomms11250 (PMC4829659; doi:10.1038/ncomms11250)
Supplement: Supplementary Information — Supplementary Figures 1-25, Supplementary Table 1, Supplementary Notes and Supplementary References. [file ncomms11250-s1.pdf]

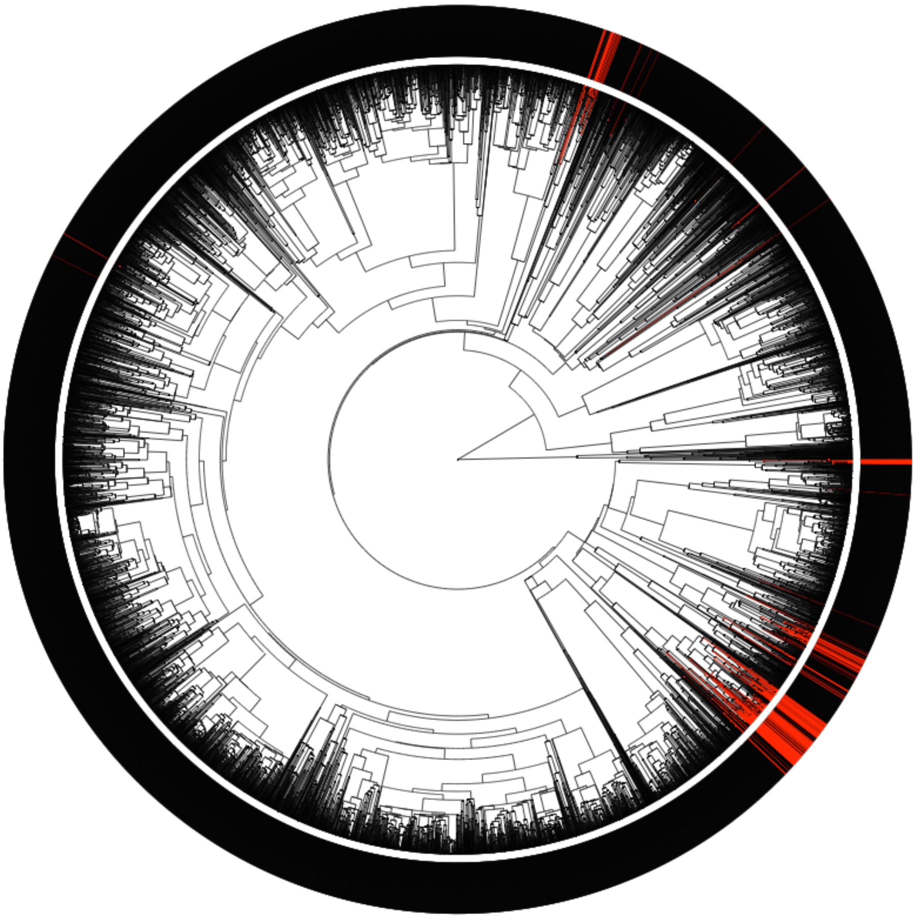

Supplementary Figure 1: Distribution of carnivore species within the Jetz et al. phylogeny. The phylogeny presented here was randomly sampled from the 10000 trees from the collection using the Ericson backbone.

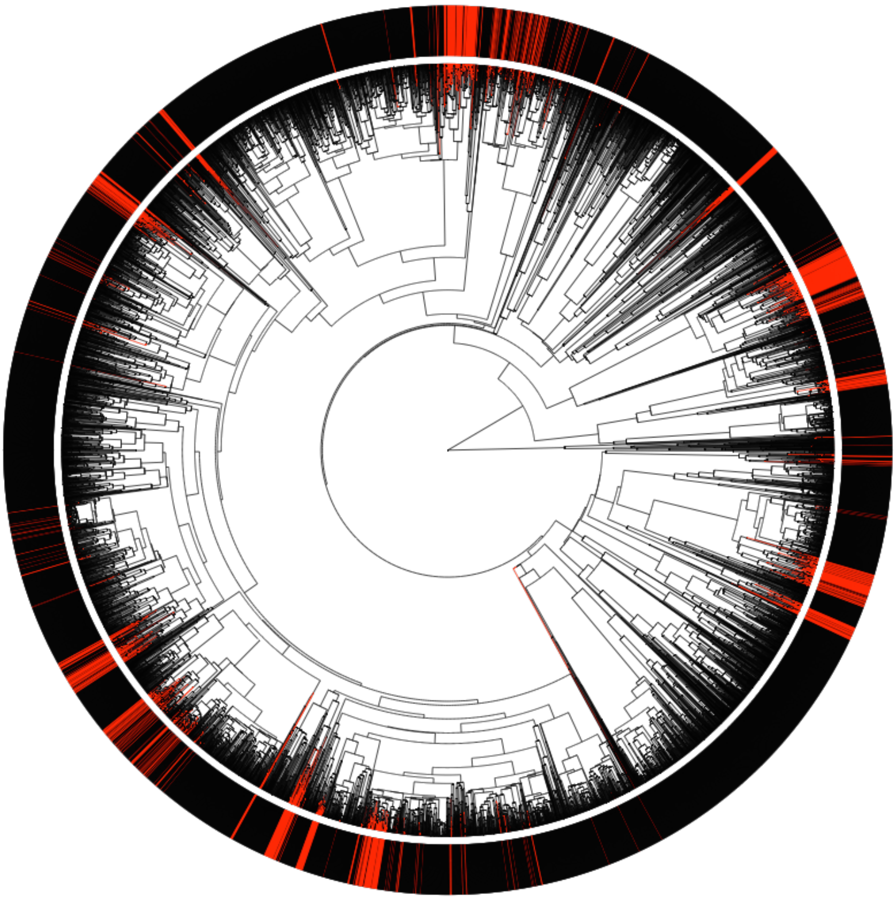

Supplementary Figure 2: Distribution of frugivore species within the Jetz et al. phylogeny. The phylogeny presented here was randomly sampled from the 10000 trees from the collection using the Ericson backbone.

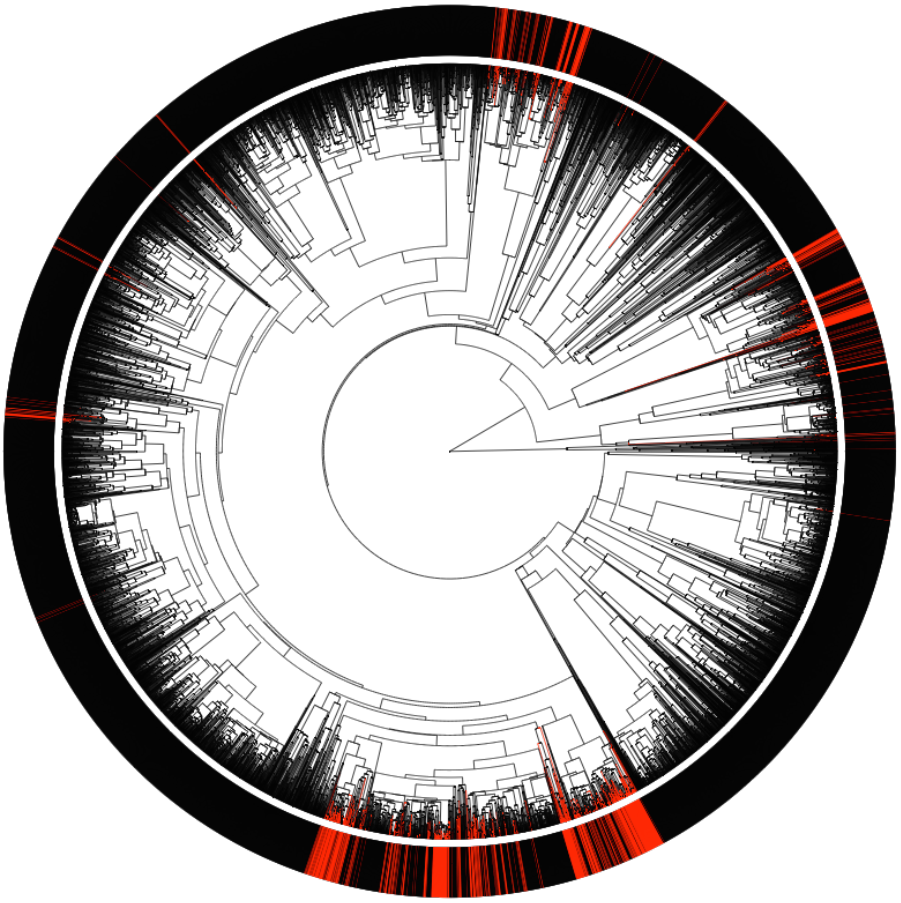

Supplementary Figure 3: Distribution of granivore species within the Jetz et al. phylogeny. The phylogeny presented here was randomly sampled from the 10000 trees from the collection using the Ericson backbone.

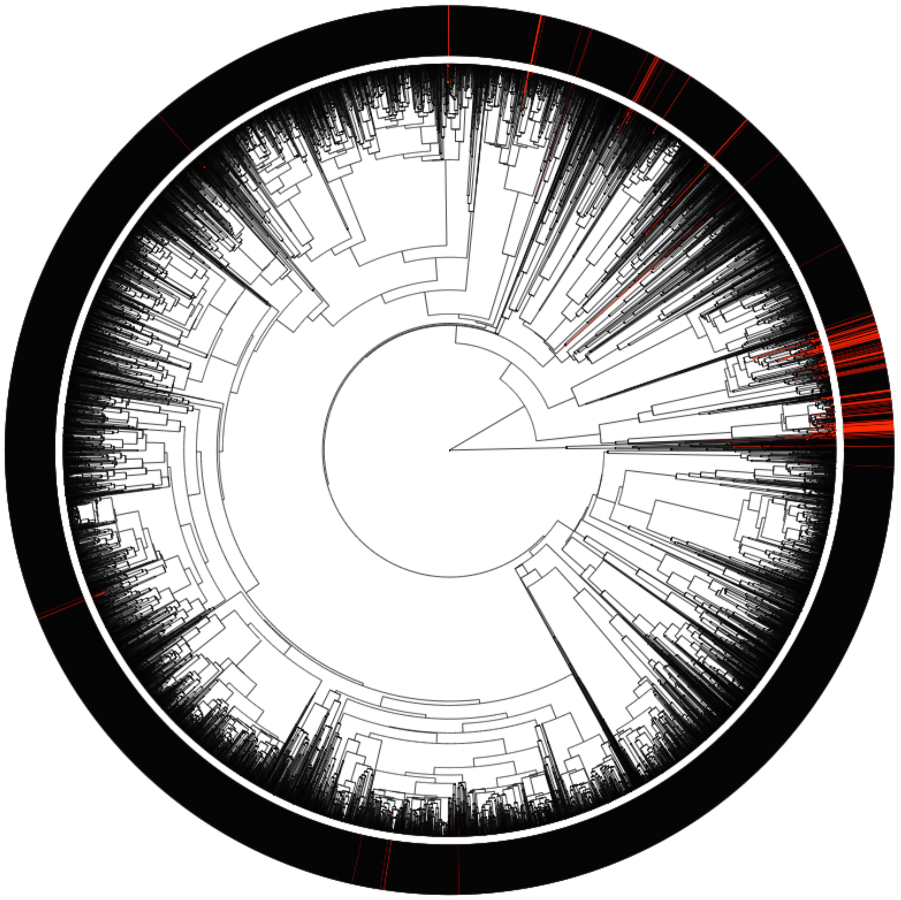

Supplementary Figure 4: Distribution of herbivore species within the Jetz et al. phylogeny. The phylogeny presented here was randomly sampled from the 10000 trees from the collection using the Ericson backbone.

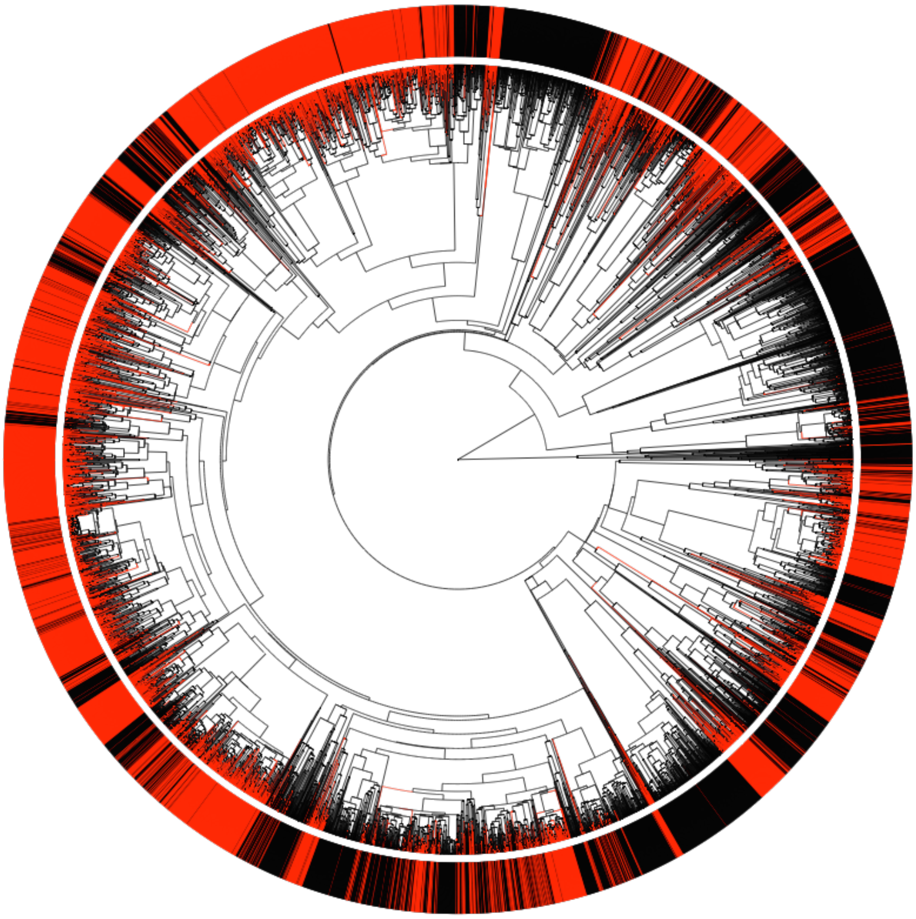

Supplementary Figure 5: Distribution of insectivore species within the Jetz et al. phylogeny. The phylogeny presented here was randomly sampled from the 10000 trees from the collection using the Ericson backbone.

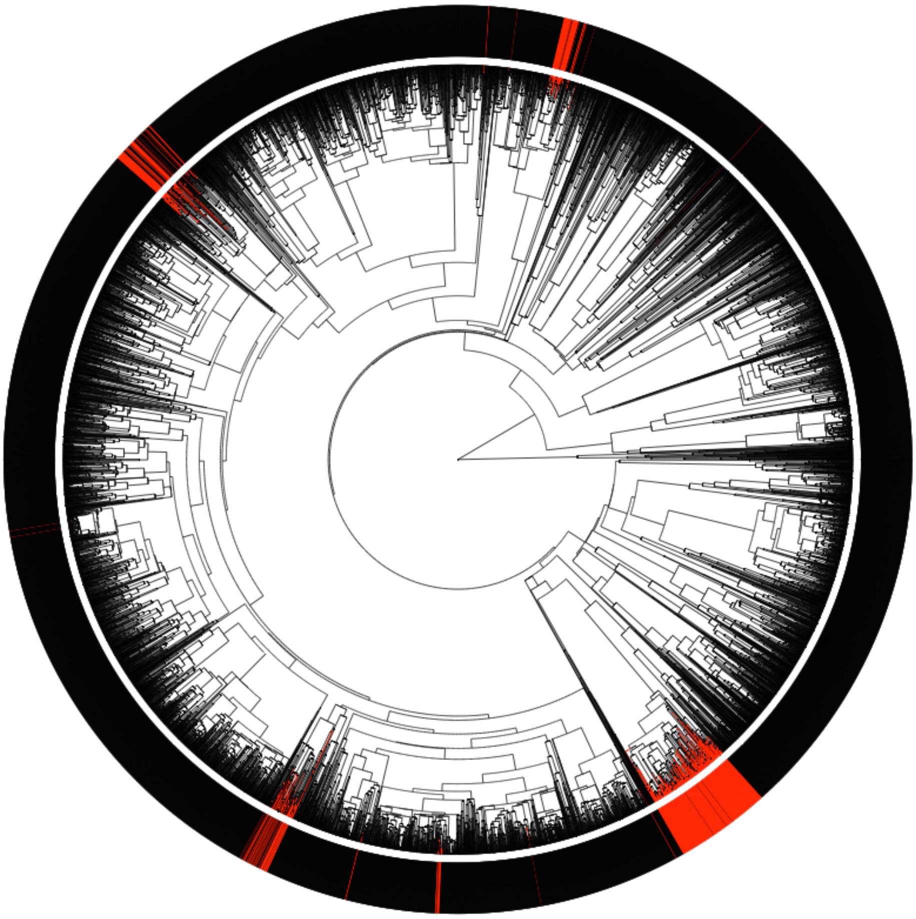

Supplementary Figure 6: Distribution of nectarivore species within the Jetz et al. phylogeny. The phylogeny presented here was randomly sampled from the 10000 trees from the collection using the Ericson backbone.

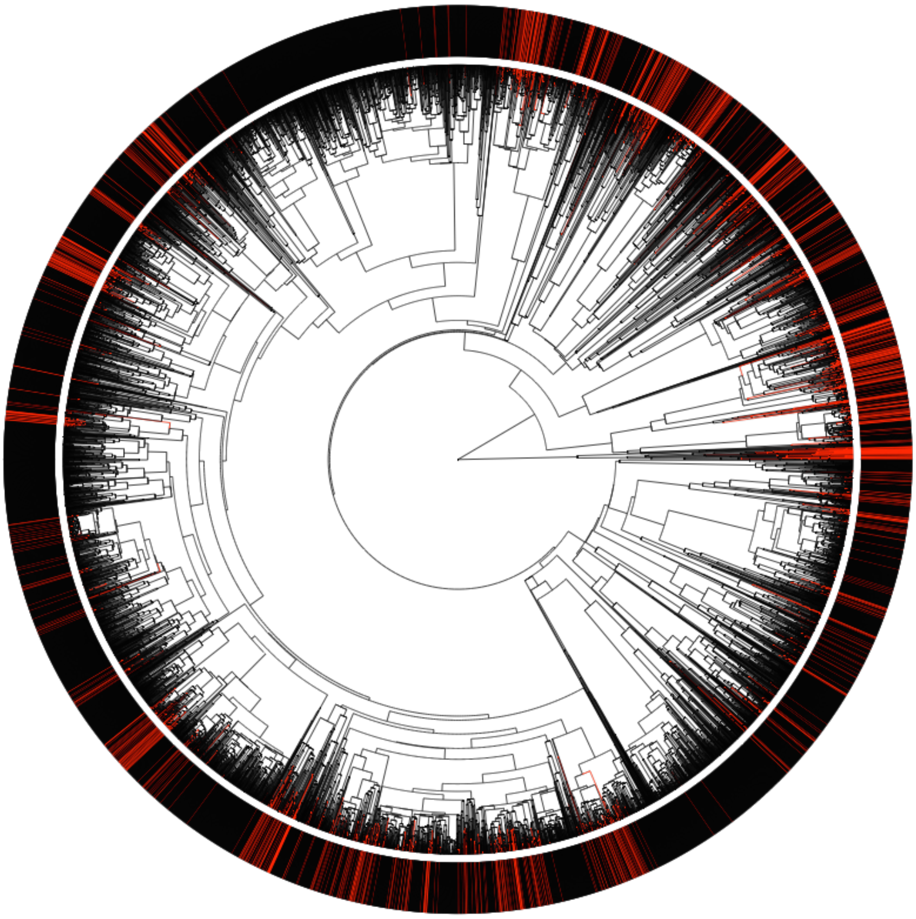

Supplementary Figure 7: Distribution of omnivore species within the Jetz et al. phylogeny. The phylogeny presented here was randomly sampled from the 10000 trees from the collection using the Ericson backbone.

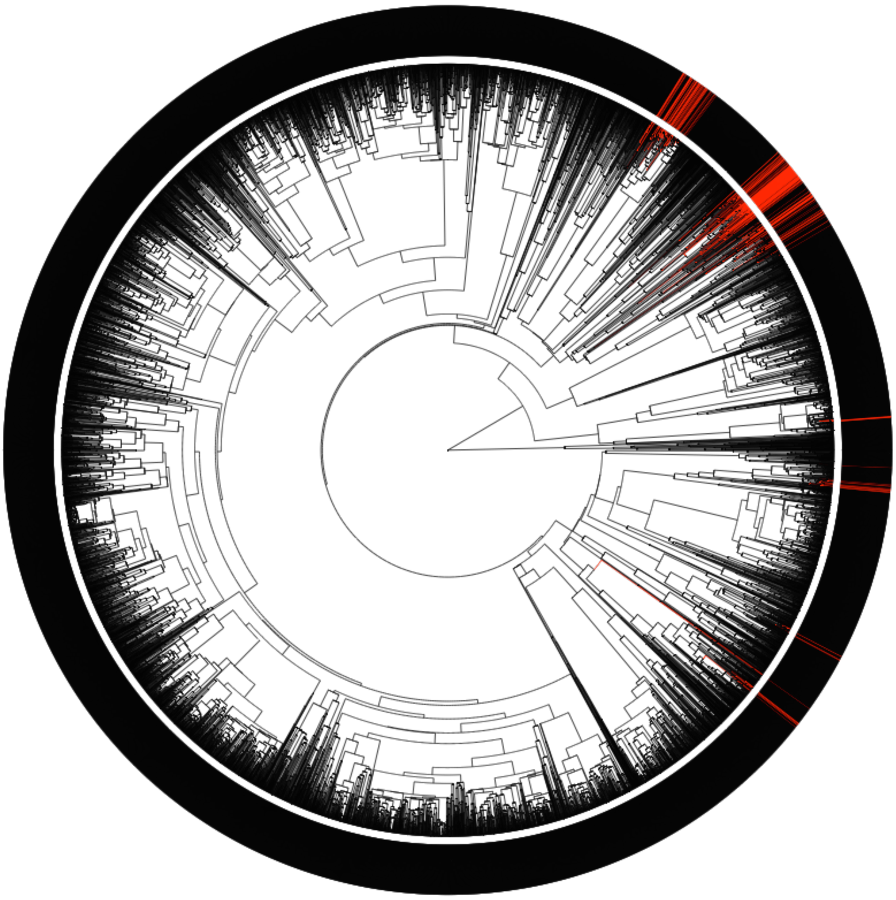

Supplementary Figure 8: Distribution of piscivore species within the Jetz et al. phylogeny. The phylogeny presented here was randomly sampled from the 10000 trees from the collection using the Ericson backbone.

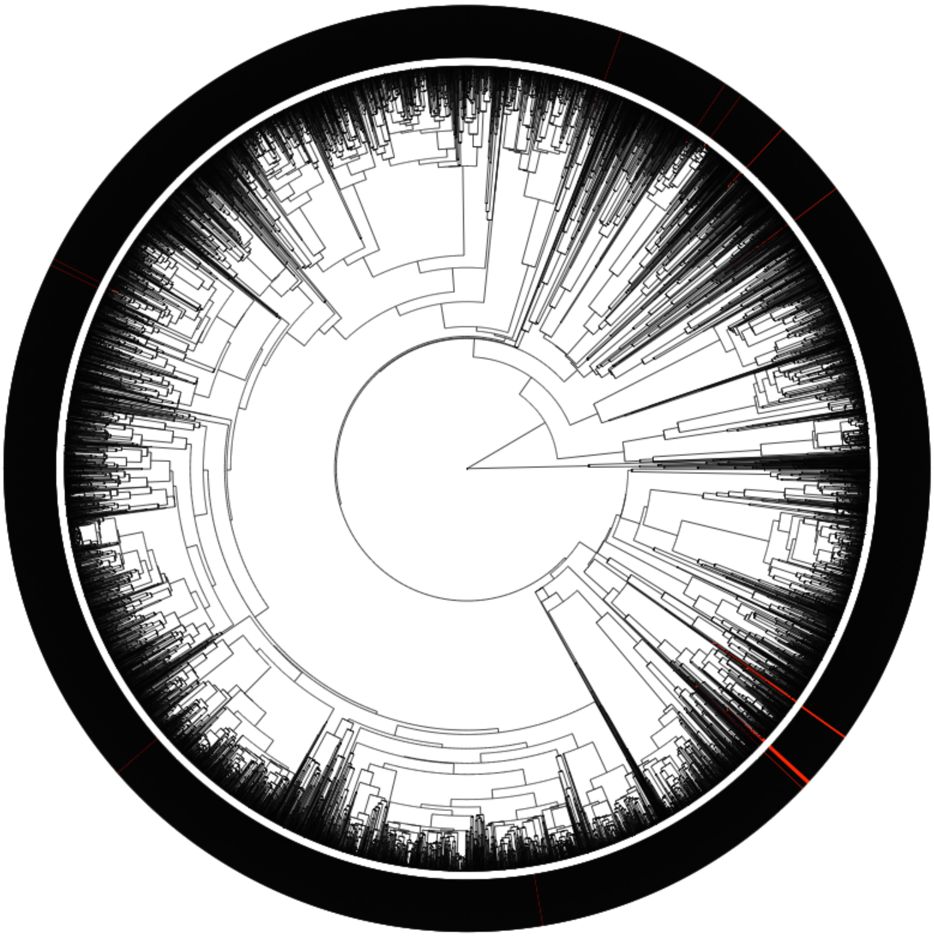

Supplementary Figure 9: Distribution of scavenger species within the Jetz et al. phylogeny. The phylogeny presented here was randomly sampled from the 10000 trees from the collection using the Ericson backbone.

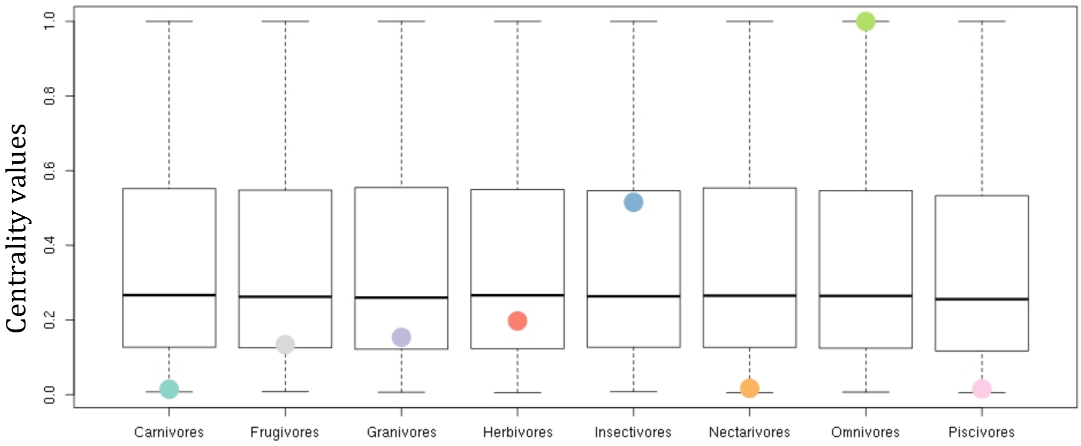

Supplementary Figure 10: Boxplots of null distributions of centrality values for each node in the transition network. The dots represent the calculated values for the data. Horizontal lines represent mean values, whereas the intervals indicated by dashed lines and ticks represent 1.5 times the interquartile range from the first (lower limit) and third (upper limit) quartiles.

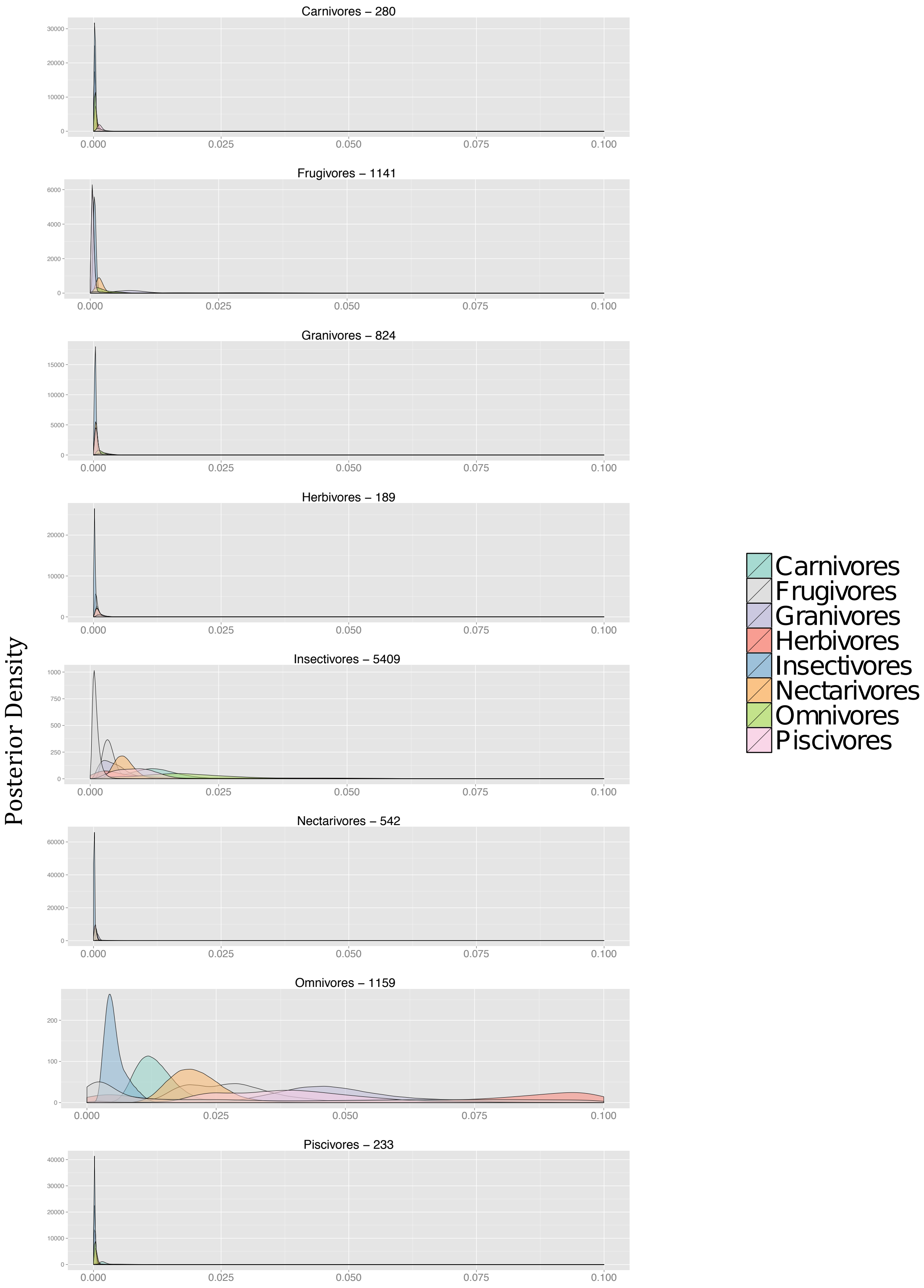

Supplementary Figure 11: Posterior distributions of transition rates into each one of the diets coming from all other guilds. X-axis is truncated for better visualization.

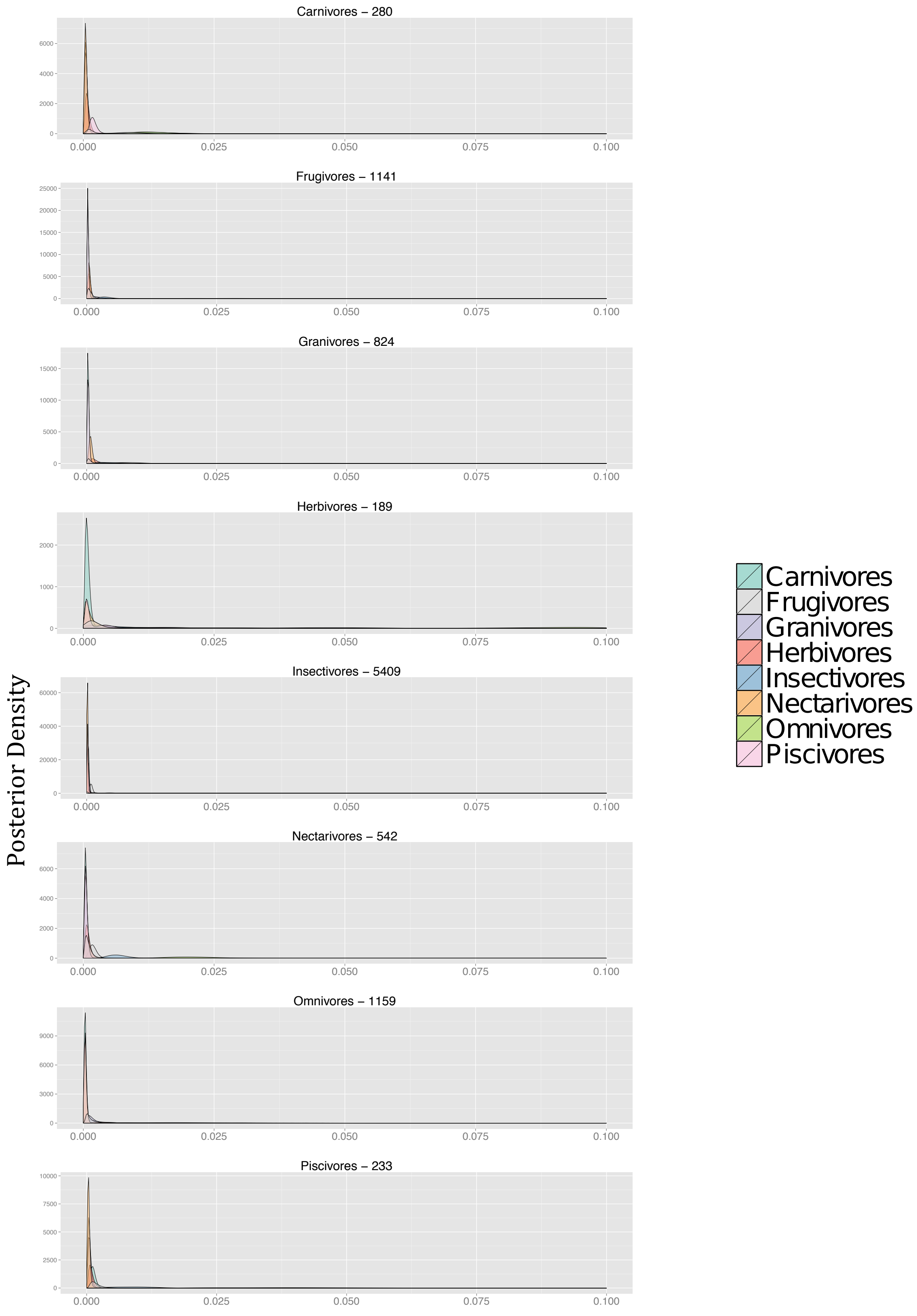

Supplementary Figure 12: Posterior distributions of transition rates from each one of the diets going into all other guilds. X-axis is truncated for better visualization

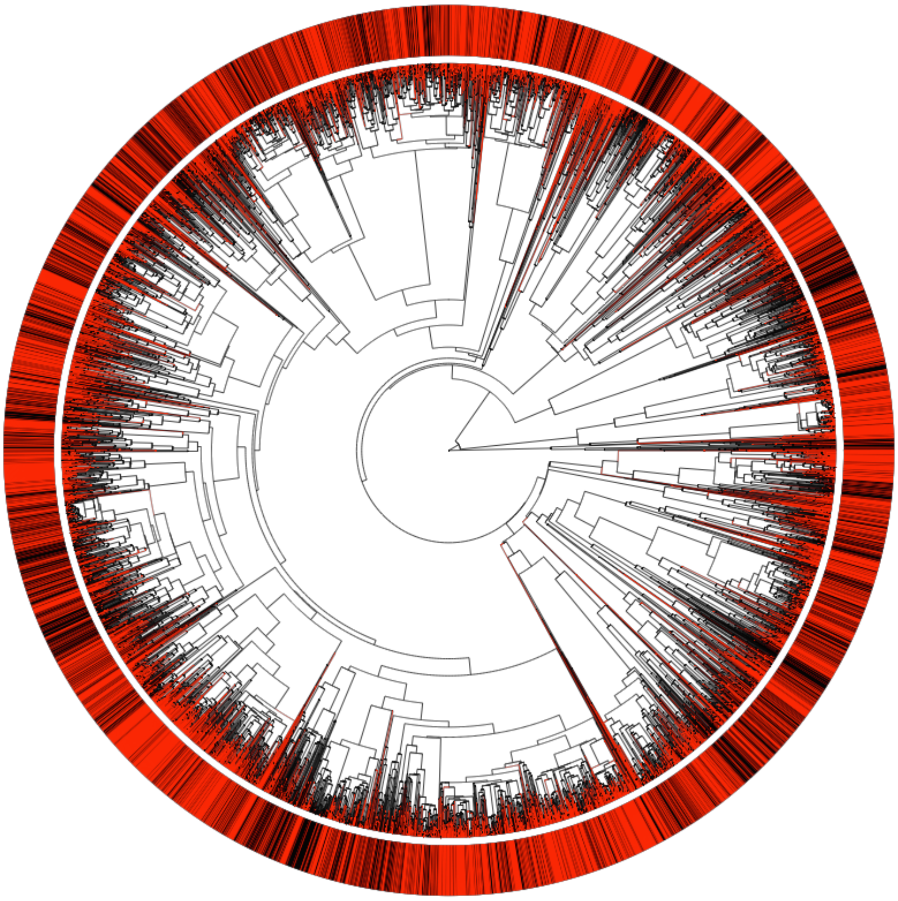

Supplementary Figure 13: Distribution of species without molecular data (3323 species, in black) and with molecular data (6670 species, red). The species without molecular data were inserted into the molecular phylogeny through a Yule process.

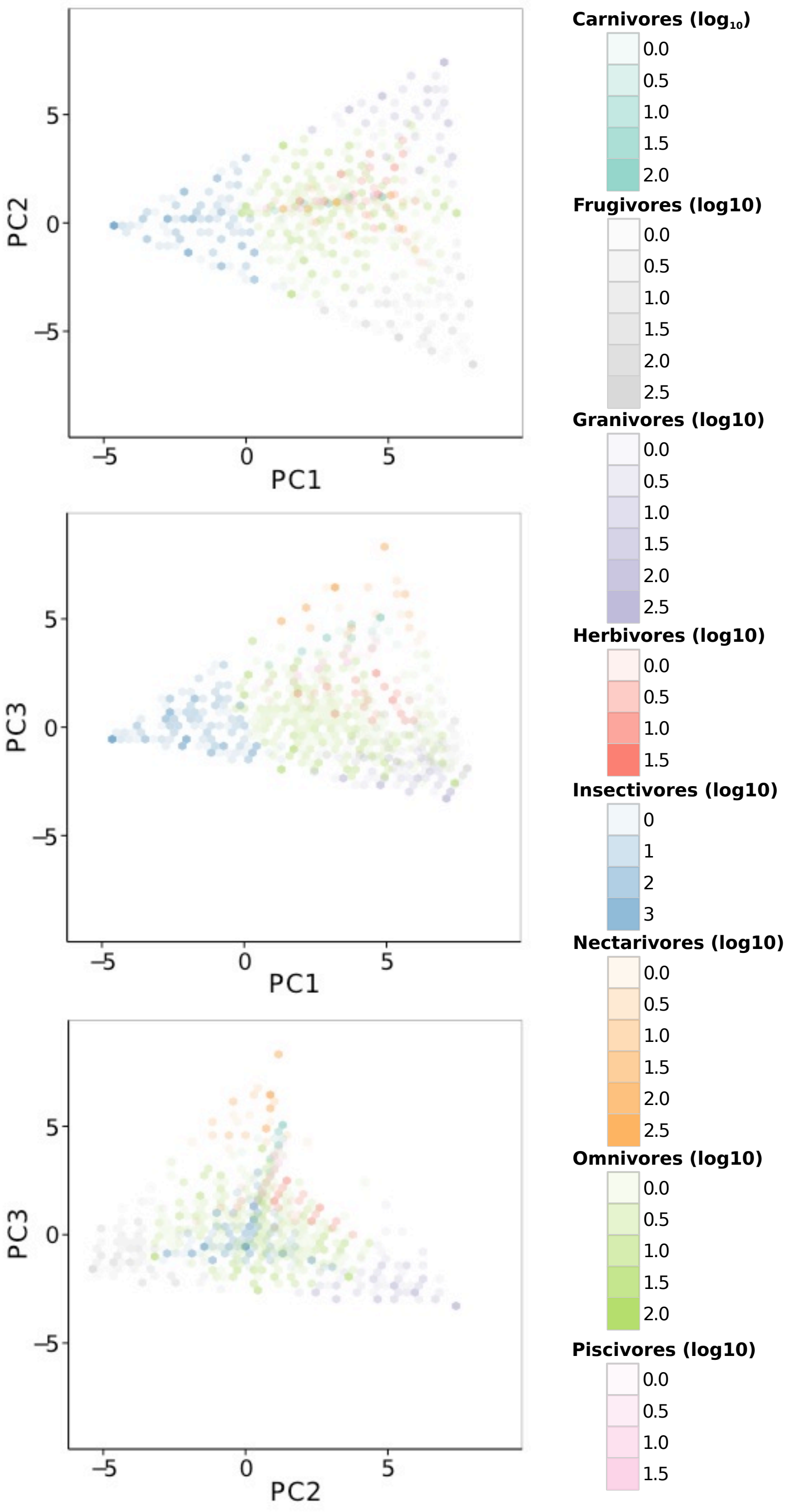

Supplementary Figure 14: First 3 principal components of PCA using the scores of each food item for omnivore species as variables plotted against one another. It is possible to see that herbivores and piscivores are similar to omnivores in all combinations, potentially reflecting the high transition rates from these two guilds into omnivores. Also possible to see that omnivory overlaps with more than 2 guilds in all axes.

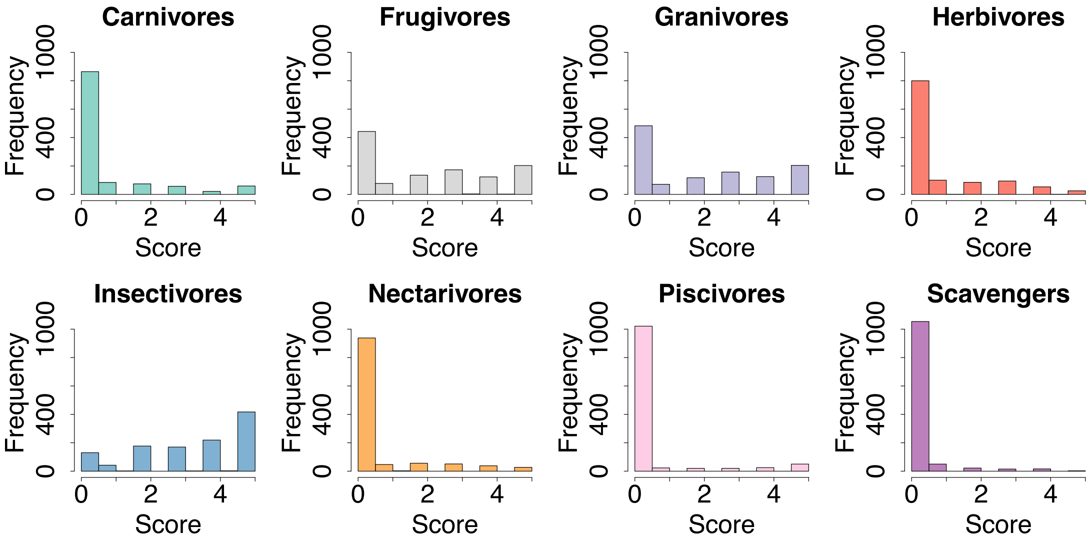

Supplementary Figure 15: Dietary composition of omnivorous birds (n = 1158 species). Frequency distribution of scores for each food item in the diet of all.

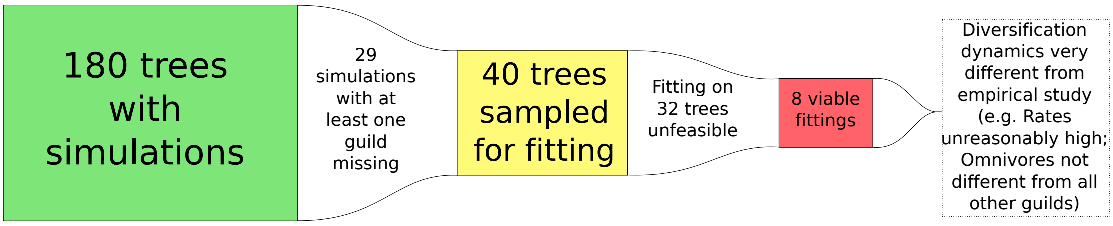

Supplementary Figure 16: Flowchart indicating the simulation protocol. Simulated scenarios where rates of diversification were not associated with trait states were unable to produce simulations that were similar to empirical data. Even with the 8 viable simulations for fitting, we could not observe any similarity between those fittings and the empirical results (posterior distributions do not show omnivores [or any other guild] as having negative net diversification rates, nor they show one guild with clearly distinct rates). The lack of viable simulations and differences in rate estimates and trait state distributions indicate that without an associated speciation and extinction dynamics the transition rates alone together with the empirical tree topologies cannot recover the observed empirical patterns.

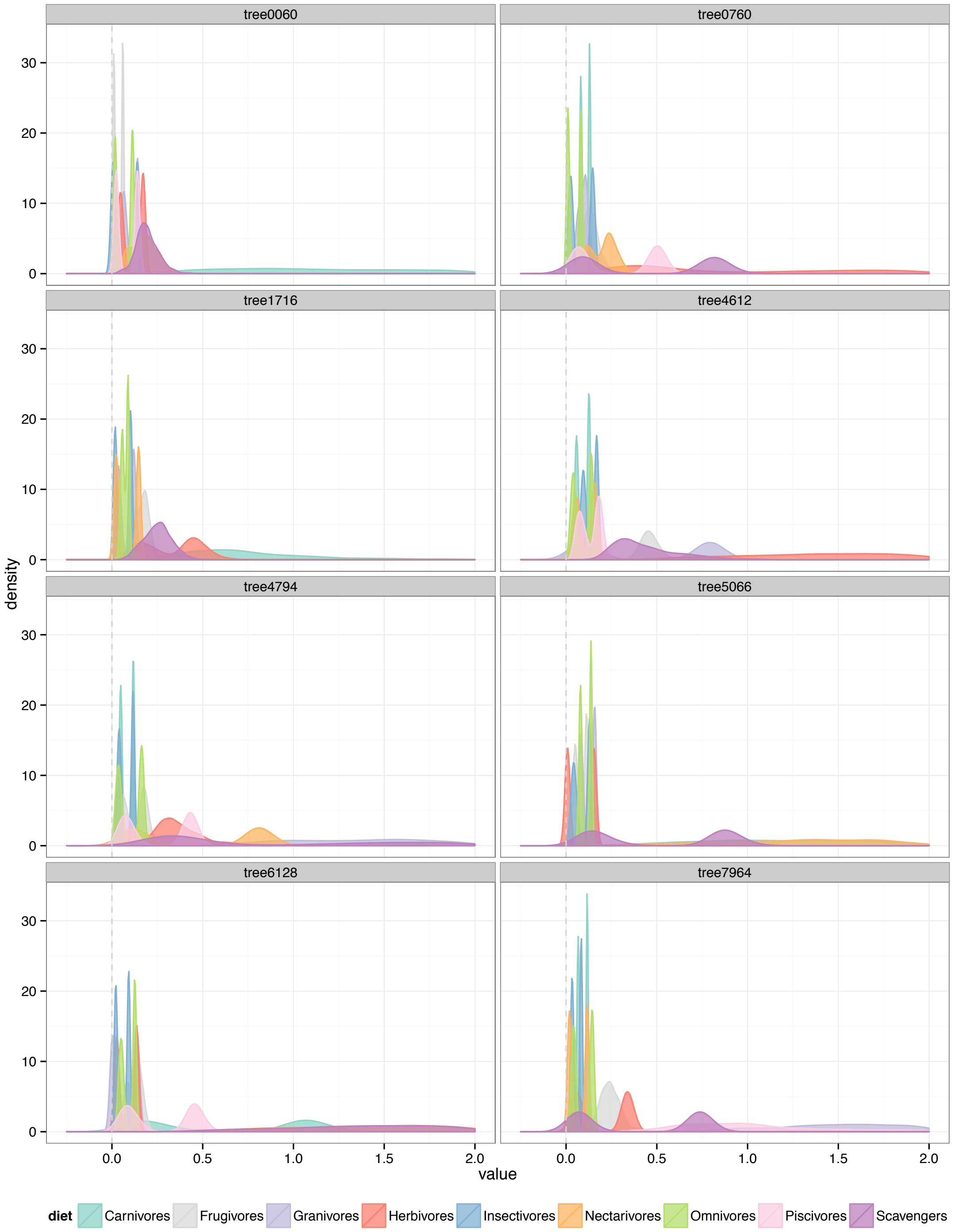

Supplementary Figure 17: Net diversification rates for all guilds in each of the 8 remaining simulations of character evolution using empirical transition rates. In none of the simulations it is possible to observe omnivores showing a distinct evolutionary dynamics in relation to all other guilds. Moreover, no dietary guild showed a predominantly negative net diversification as seen in the empirical data for omnivores.

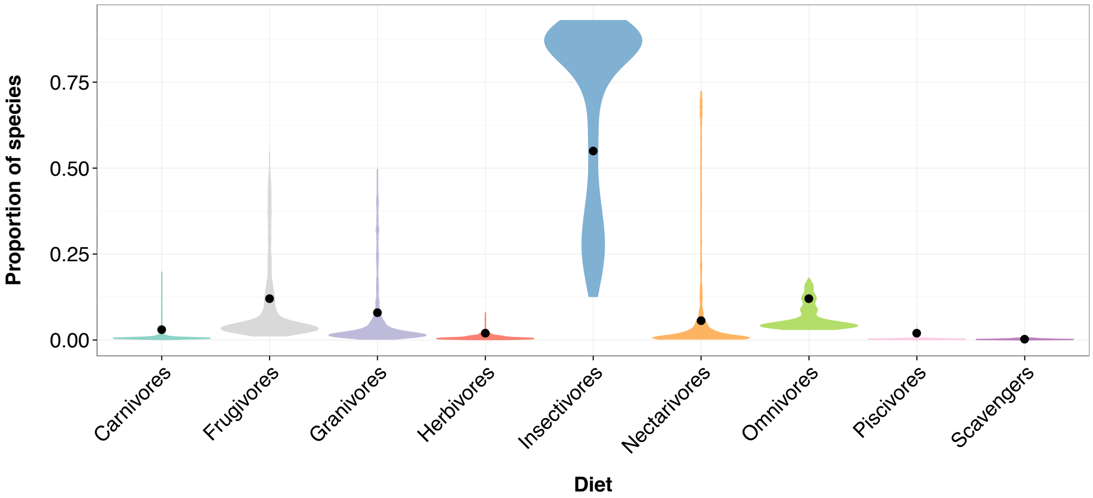

Supplementary Figure18: Posterior predictive distributions for model adequacy. Distribution of proportions that each diet represents in the 1000 simulated trees using estimated empirical rates. The black dots represent the proportion of diets present on the data used in the study. Simulations produced proportions that encompass the empirical values. Although the figure scale is not adequate to see all the proportion distribution simultaneously, the empirical piscivores proportion is within the range, close to the upper limit of simulated proportions .

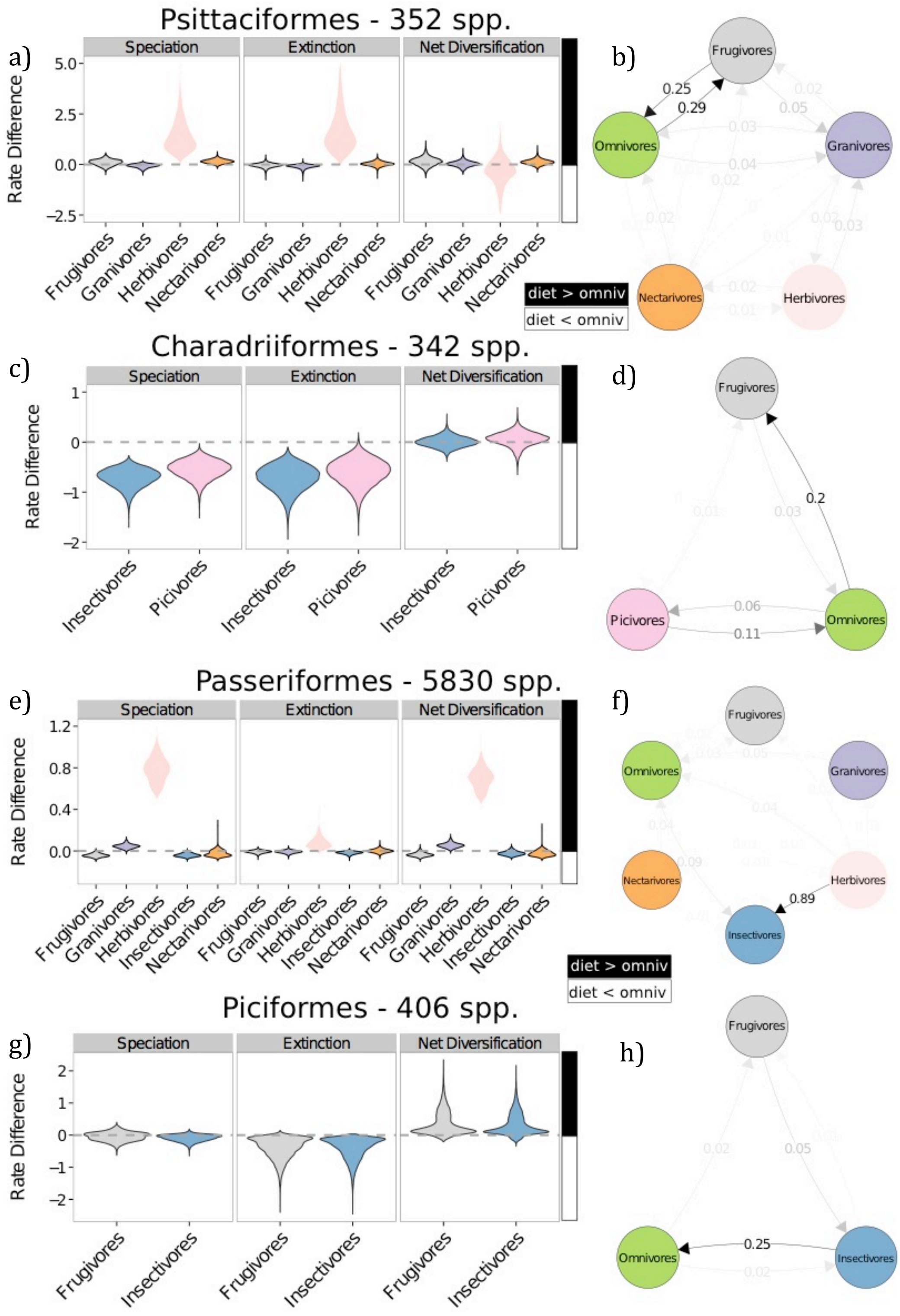

Supplementary Figure 19: Posterior distributions of speciation, extinction and net diversification rates (panels a, c, e, and g) and transition network (panels b, d, f, and h) for each of the 4 analyzed subclades: a-b) Passeriformes; c-d) Piciformes; e-f) Psittaciformes; g-h) Charadriiformes. Extinction rates show a similar trend to the main study, usually being higher in omnivores, whereas speciation and net diversification rates show inconclusive patterns. Transition networks for Charadriiformes (h) show a pattern similar to the whole tree, while the other three networks show different patterns.

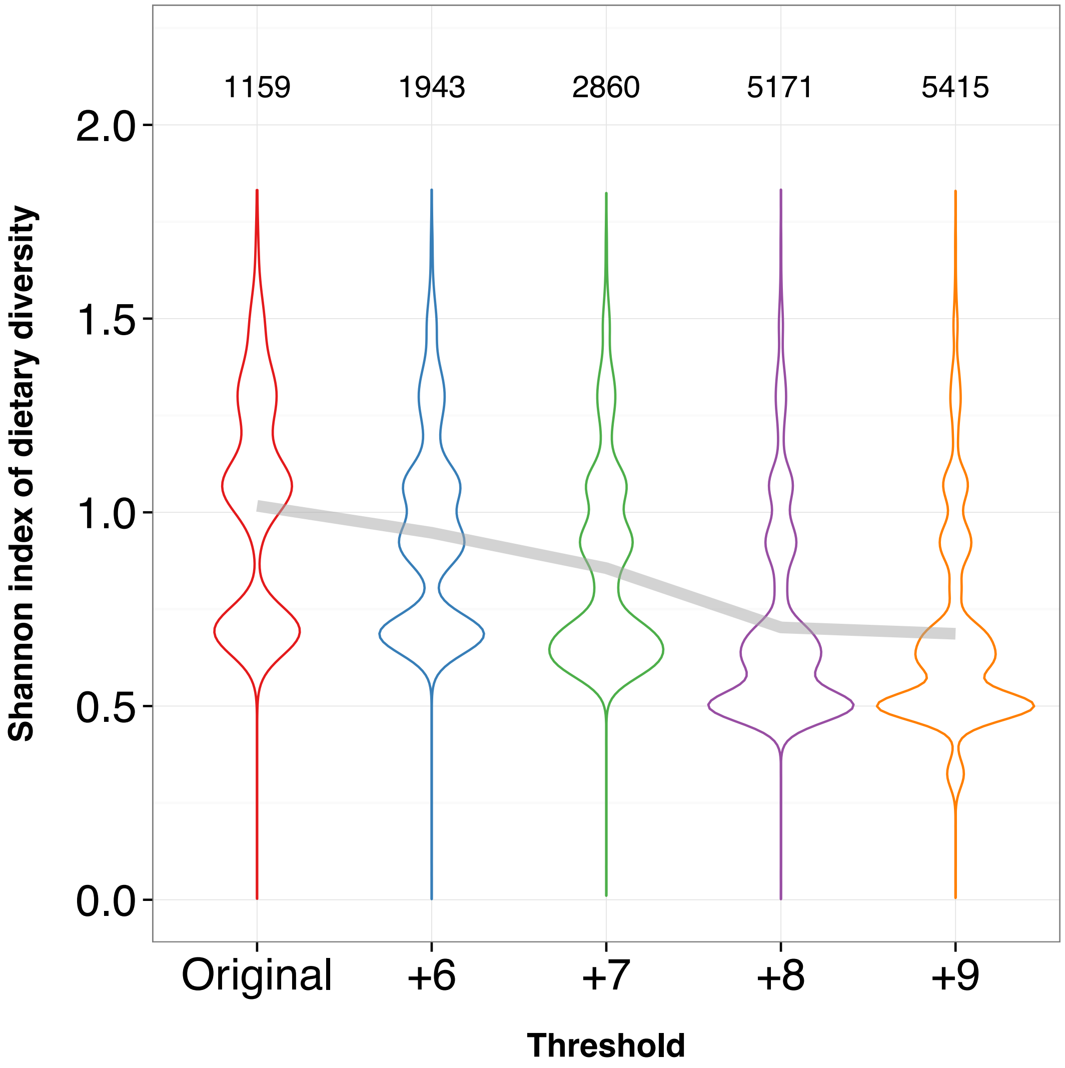

Supplementary Figure 20: Distribution of dietary diversity indexes for omnivores classified using different classification schemes. The number on the top represent the number of species classified as omnivores in each scheme. It is possible to see a steeper decrease in the average index when increasing the threshold from 70% to 80%. This reinforces our criterion in using 50% as an upper limit for a given item to be consumed in the diet of an omnivore species.

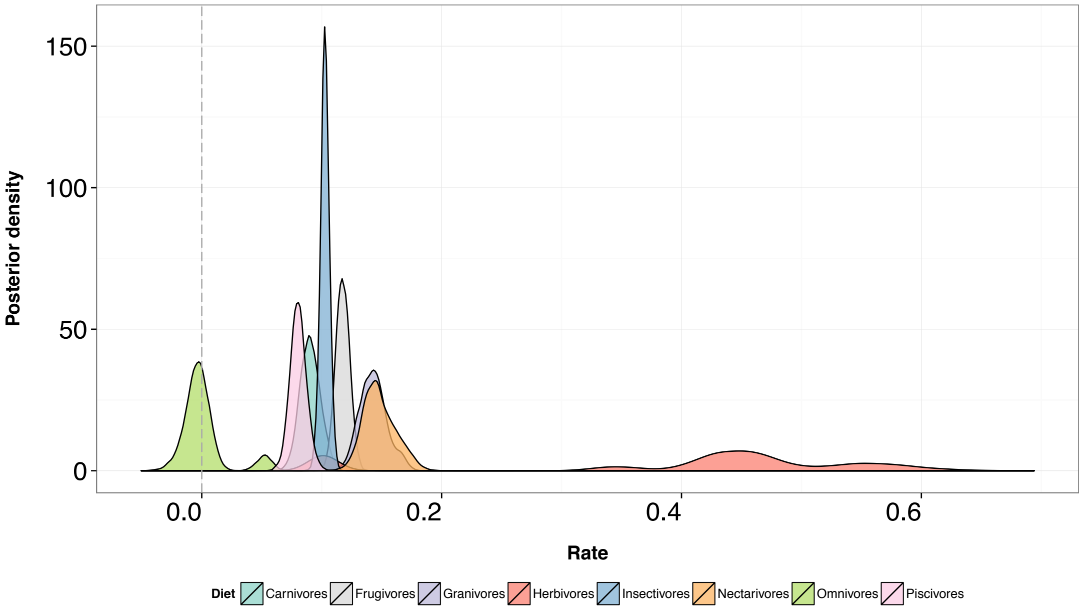

Supplementary Figure 21: Combined posterior distributions of net diversification rates for each dietary guild using the new classification scheme (70% threshold). Omnivores still have lower net diversification rates when compared to all other guilds.

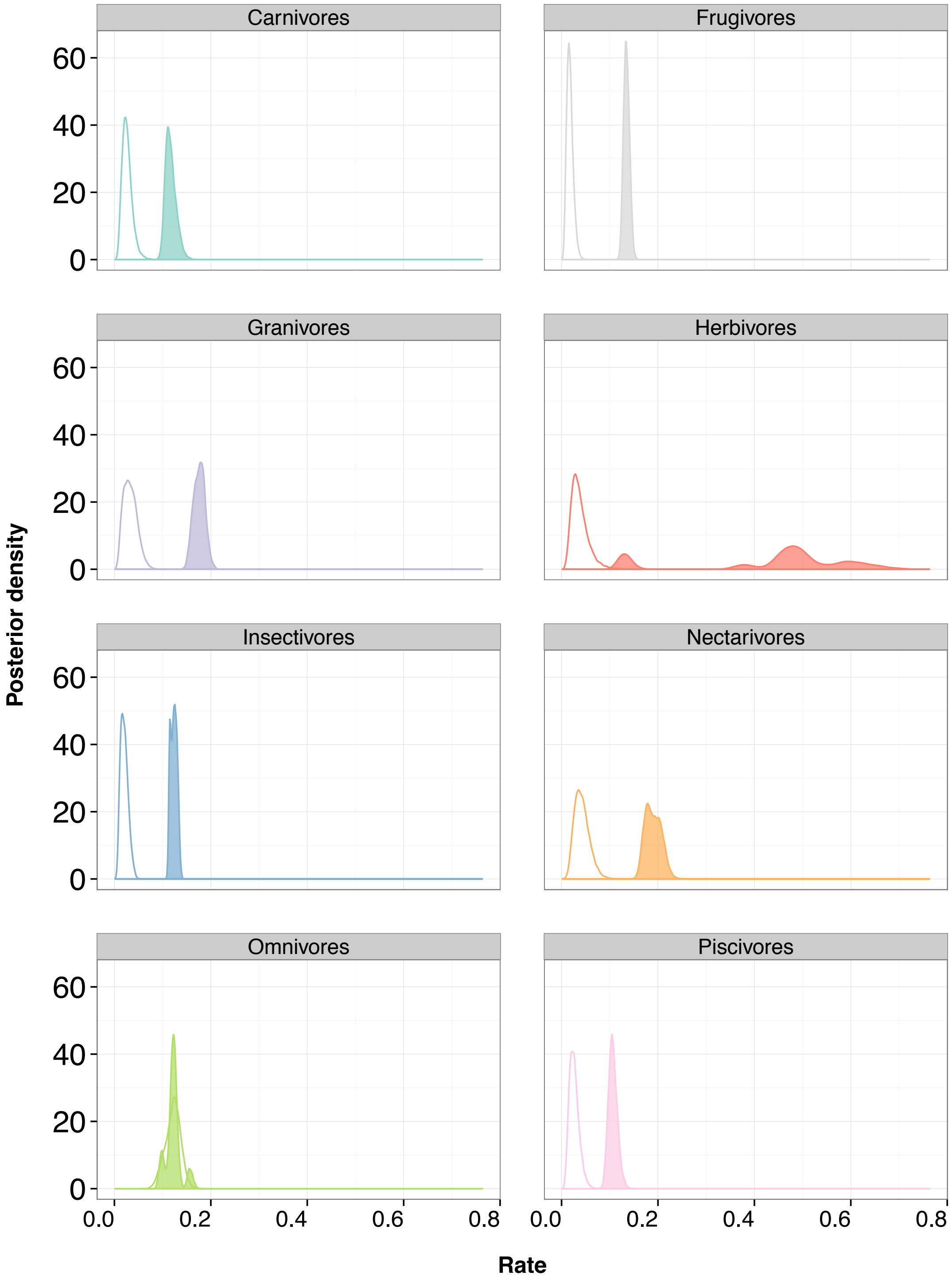

Supplementary Figure 22: Combined posterior distributions of speciation (filled curves) and extinction (hollow curves) rates for each guild using the new classification scheme (70% threshold). Omnivores is the only guild in which there is a almost complete overlap between the curves of the two rates.

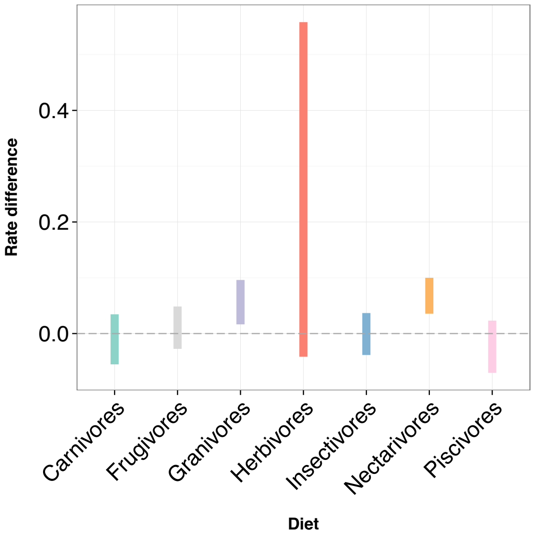

a)

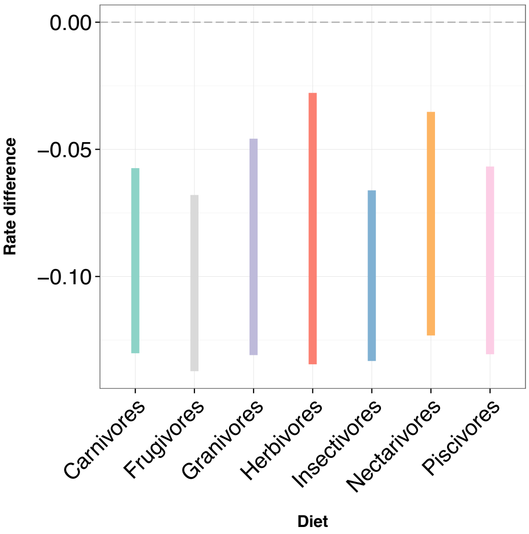

b)

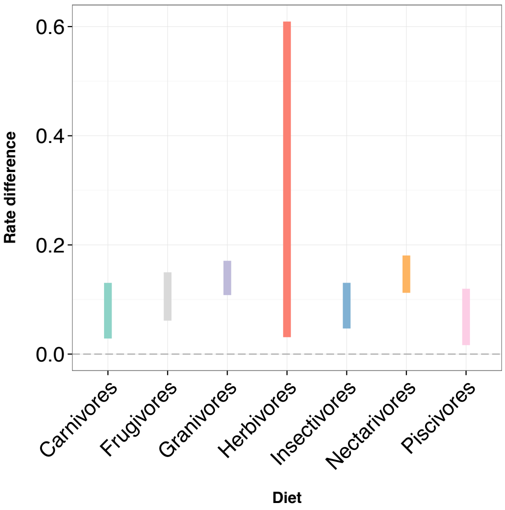

c)

Supplementary Figure 23: 95% credibility intervals for the posterior distributions of differences between speciation (a), extinction (b) and net diversification (c) of all guilds in relation to omnivores using the new classification scheme (70% threshold). Omnivores still show either smaller speciation and/or greater extinction rates when compared to other guilds, which results in an overall low net diversification.

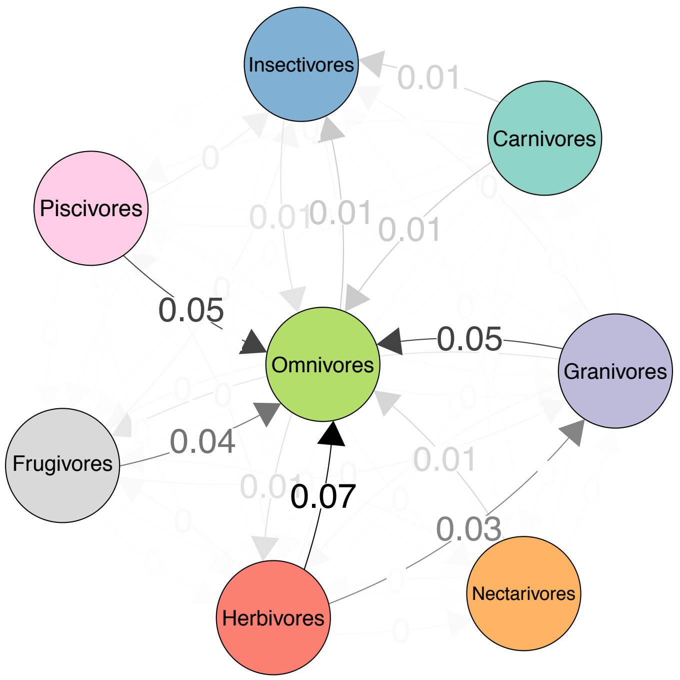

Supplementary Figure 24: Network of estimated transition rates between diets using the new classification scheme (70% threshold). Omnivores keep being the preferential target in transitions even in this new classification scheme, reinforcing their role as macroevolutionary sinks.

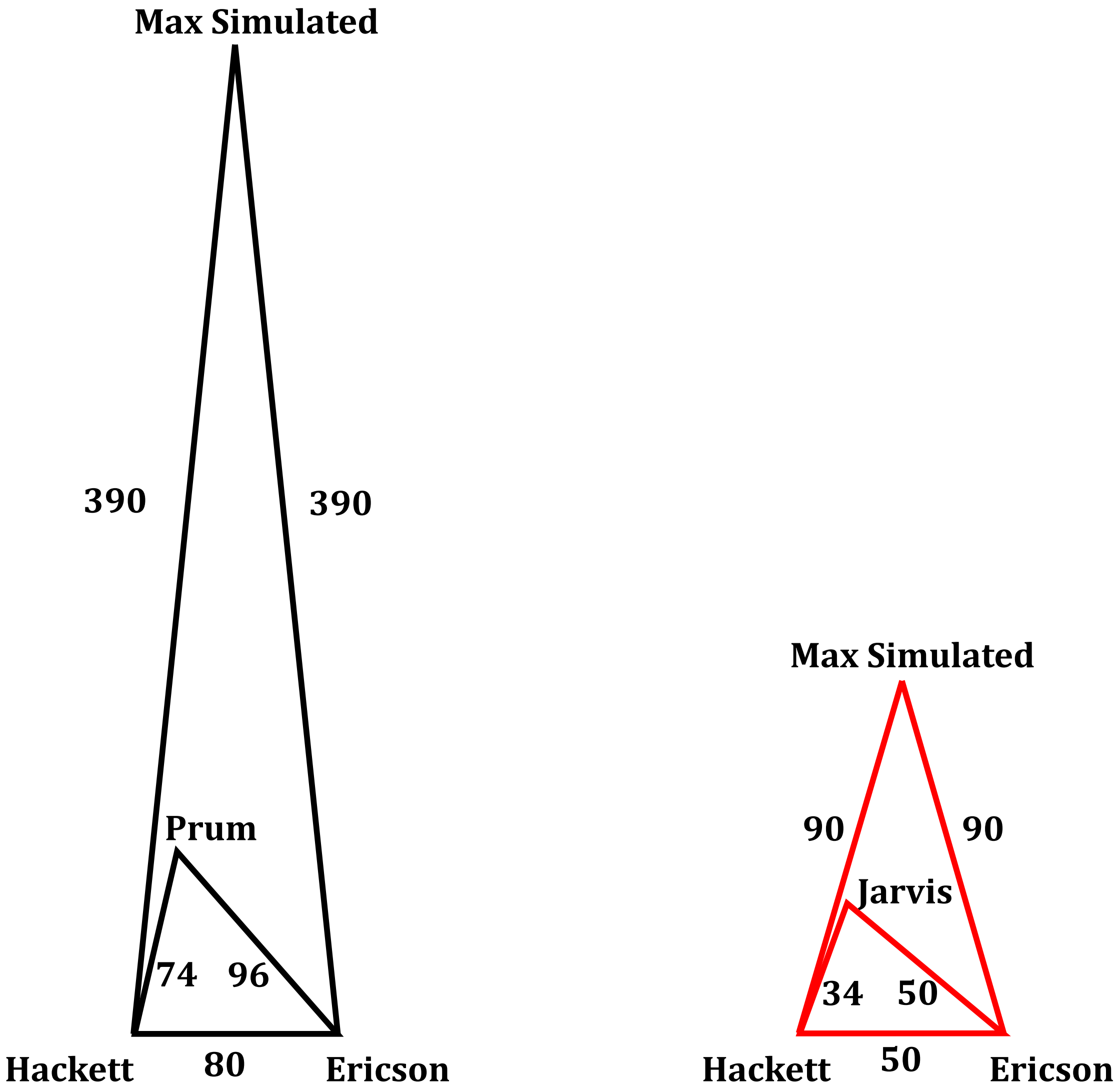

Supplementary Figure 25: Representation of the Robinson-Foulds distances between the two backbone trees (Hackett and Ericson, as used in our work) and two recently published high-order phylogenies (Prum, Javis) as well as simulated trees (Max Simulated). The distance values represent the number of permutations needed to transform one tree into another tree. The results show that the relative difference between the two backbones is similar to the difference between each backbone and either one of the two new phylogenetic hypotheses. This suggests that phylogenetic heterogeneity (or uncertainty) as incorporated in our analysis (the use of trees from both backbones) encapsulates an amount of phylogenetic dissimilarity that is comparable to the one captured when comparing the backbone trees with other phylogenetic hypothesis.

Supplementary Tables

Supplementary Table 1: Number of simulations with each ancestral state

Diet	Number of simulated trees
Carnivores	30
Frugivores	120
Granivores	80
Herbivores	20
Insectivores	550
Nectarivores	57
Omnivores	120
Piscivores	20
Scavengers	3

Supplementary Notes

A1) Assessing the coupling between speciation/extinction and transition rates

Our results indicate that 29 out of the 180 simulations had at least one guild missing. The considerable percentage of simulations with missing guilds (approximately 16%) is already indicative that transition dynamics alone (without a coupled speciation and extinction dynamics) rarely reproduce the empirical distribution of diets observed in extant species.

From the 40 randomly selected simulations that contained all guilds, our results showed that 32 out of those 40 simulations where we fitted MuSSE did not provide reliable results. The MCMC chains for those took as much as 20 times longer than the viable ones to reach the same number of iterations and showed no sign of convergence. This poor performance for those MCMC chains is caused by recurrent unrealistic estimated rate values such as speciation rates of 10^4 species per lineage per million years. This is a reflection of underrepresentation of some guilds, which renders the estimate unreliable. Again, this lack of

convergence can be interpreted as evidence that transition rates alone are not capable to generate scenarios similar to the one seen in the empirical tree.

Taken together, those two previous results suggest that when simulations ignored the rates of speciation and extinction associated with trait states, the vast majority of these simulations resulted in scenarios, which differed from the empirical data either in relation to the proportion between guilds and/or from the perspective of rate estimates, to the point that those were not used or had to be discarded to the fitting exercise (Supplementary Figure16).

The fitting of MuSSE was only possible on 8 simulations and in none of these remaining simulations we observe negative diversification rates for omnivores (Supplementary Figure 17). In fact, no trait state was estimated to be associated with negative net diversification rates on those 8 simulations, and the rate estimates were not even vaguely similar to the empirical estimates. With respect to our empirical case, these simulation results suggest that the transition dynamics (without changes in speciation and extinction due to trait states) and the specific tree topology alone are not capable of reproducing the empirical diversification dynamics (Supplementary Figure 17). Thus, we suggest that our results are unlikely to be caused by model misbehavior, but rather by an association between trait states (diets) and diversification rates.

A2) Posterior predictive simulations to test for model adequacy

The results from the simulation that used the empirical rate estimates associated with different dietary guilds produced a distribution of proportions between guilds that lies around the proportions observed in the empirical data (Supplementary Figure 18). This indicates that the rates estimated by our analysis can generate plausible scenarios (Supplementary Figure 18). We interpret this result as evidence that the models used to characterize the diversification dynamics associated with the different dietary guilds are adequate.

A3) Model fitting in sub-clades

The results from the sub-clades analyses showed a similar pattern for the extinction regimes associated with omnivory. Here, as in the main analysis of the

whole tree, extinction rates associated with omnivory tended to be either indistinguishable from other guilds or higher than in other guilds (Supplementary Figure 19). The exceptions were the extinction rates of herbivores in both Passeriformes (Supplementary Figure 19.a) and Psittaciformes (Supplementary Figure 19.c). We suggest that these very high extinction rate estimates for herbivores result from the small number of herbivore species present in each of those orders (12 species in Passeriformes and 10 species in Psittaciformes) and therefore are in fact unreliable estimates.

Supplementary Figure 19 also shows that estimates of speciation rates of omnivores are either not significantly different or higher than the speciation rates of other guilds for all four orders (Supplementary Figure 19.a,c,e,g). This is different from what we discovered in the main analysis, but we note that the transition dynamics between guilds are also different from the dynamics estimated for the whole tree with the exception of Piciformes, which show transitions towards omnivory. We suspect that using sub-clades will lead the models to preferentially assign the origin of new omnivore species to speciation rather than to transitions because fewer transitions remain at the sub-clade level.

Although these sub-clade results indicate some differences to the whole tree analysis, the direct interpretation of the sub-clade analysis in xxSSE models other than BiSSE may not be as straightforward as previously advocated for BiSSE (68). For a binary-state character all sub-clades of a given tree will have the same number of states (two) as the whole tree, but when working with multi-state characters this might not be necessarily true (for example the order Passeriformes comprise more than half of all bird species, but no carnivore, piscivore or scavenger species are present). Second, as previously mentioned, dividing a tree into sub-clades might obscure the dynamics of transitions, because a state that might have had multiple origins in the whole tree might be perceived as having few transitions in a sub-clade that has, for example, just one or very few transitions. Finally, when looking at sub-clades some states may be very under-represented. According to (1), the low relative number of species in a given state leads BiSSE models to incorrectly estimate the parameters. This might be also the case in other xxSSE models, especially when the absolute

number of species is low (for example the very low number of herbivore species in both Passeriformes and Psittaciformes, i.e. 12 and 10, respectively). Given those constraints, we do not expect that the sub-clade analysis always replicates the results from the whole tree analysis. Therefore, the results from this analysis should be taken with caution when using a MuSSE model.

A.4) General conclusion on the use of MuSSE in our data.

To conclude, the first two sets of tests provided good evidence for the proper behavior of the model to estimate true state-dependent speciation, extinction and transition rates. The results from the sub-clade analysis were rather inconclusive, although some trends (e.g. extinction regimes) were similar to the whole tree analysis. We suggest that the overall signal from the sensitivity analyses provides evidence that the model is providing reliable results, and that the qualitative results and conclusions drawn from the whole tree analysis are robust.

B) Sensitivity analysis for dietary classification

The results shown in supplementary figures 20-24 suggest that a more inclusive categorization of omnivory would result in the same patterns as observed in the main analysis. In this new classification scheme, omnivores also showed either lower speciation and/or higher extinction rates when compared to all other dietary guilds. This ultimately results in a lower (and again even negative) net diversification rate for this guild. Additionally, the observed transition rate patterns are similar to what we recover in our main analysis and support the scenario that poses omnivory as a macroevolutionary sink. We therefore suggest that our main conclusions are independent to the classification scheme used.

C) Comparison of backbone trees

It is possible to see that the two backbones used on our study show a level of phylogenetic dissimilarity that is comparable to the one captured when comparing those backbones to other recent phylogenetic hypothesis (supplementary figure 25). This suggests that phylogenetic uncertainty

incorporated in our analysis (the use of trees from both backbones) is comparable to the ones captured from the two other phylogenetic hypotheses.

Supplementary methods

A) Assessing xxSSE model limitations

We ran three sets of tests to evaluate to what extent the issues pointed out by Rabosky & Goldberg (2015) might have affected our results and conclusions. These tests were suggested by those authors as a way of diagnosing possible biases on parameter estimation due to background rate heterogeneity in phylogenetic trees. For two sets of tests (A1 and A3) that require empirical trees, we used 10 randomly sampled trees (5 from each backbone) from the 200 trees used in the study. Since the second set of tests (A2) consists of simulated trees, it does not require the use of empirical trees.

A1) Assessing the coupling between speciation/extinction and transition rates

The recent study by Rabosky & Goldberg (2015) criticizes the indiscriminate use of the xxSSE models for testing hypotheses about character state-dependent diversification rates. The main argument is that xxSSE models tend to exhibit high Type I error rates, i.e. detecting a character state-dependent diversification rate when there is no association between characters and rates ('false positives'). Given the clear relevance of such a potential problem, Rabosky & Goldberg (2015) suggested to simulate "neutral characters" on empirical trees, with the aim to investigate if xxSSE models would attribute different diversification rates for different character states even in the absence of a direct association. As an example, Rabosky & Goldberg (2015) simulated the evolution of a binary neutral character (without an influence on speciation or extinction) on a phylogeny of extant whales to quantify the Type I error rate using the BiSSE model. Their simulations involved only trait evolution (no birth-death process) and assumed symmetrical transition rates. We suggest that this way of implementation is inappropriate for testing Type I error rates in xxSSE models.

In the case of the xxSSE models, to properly test for a Type I error, the null model should be constructed with the premise that the tree itself was generated by a constant-rate birth-death process. If this is ignored, a potential false association between a simulated neutral character and rates of diversification (referred to as Type I error rates by Rabosky & Goldberg (2015)) could be either the result of model misbehavior (suggested by Rabosky & Goldberg (2015)) or result from violating the constant-rate birth-death process. The empirical trees used in our work, like most phylogenies for species-rich clades, are clearly subject to rate heterogeneity (Jetz et al 2012; Huang & Rabosky 2014) and hence deviate from the null scenario of constant-rate birth-death process. However, developing a null model that incorporates the rate heterogeneity as commonly seen in empirical trees to verify a potentially spurious association is not an easy task. Hence, rather than trying to develop a test to detect Type I errors, we think it is more appropriate to evaluate the reliability of empirical results by firstly decoupling the transition dynamics from the speciation/extinction dynamics and secondly testing whether similar associations between trait states and speciation and extinction rates can be recovered. The latter ones are ultimately the processes that determine rate heterogeneity seen in empirical trees.

We therefore propose a small modification to Rabosky & Golbderg's protocol to assess the reliability of xxSSE models when dealing with specific empirical trees. Given the intricate roles of diversification and transition rates on generating the empirical distribution of trait states on the tips, which will eventually be used to test the reliability of the xxSSE models, we suggest to control for the effect of asymmetric transition rates. This can be done by simply using the empirical transition rates instead of symmetrical transition rates when simulating trait evolution. This gives a more reliable assessment of the risk of detecting a spurious association between diversification rates and given trait states. Note that this, as in the case of Rabosky and Goldberg (2015), is not a test of Type I error in a purely statistical sense, but rather a test if the empirical pattern can be generated by a lack of association between trait states and speciation and extinction rates simulated via trait evolution on empirical trees. In the case of decoupled dynamics described above, if an association between speciation/extinction rate and trait state similar to the empirical ones is

recovered then the there is weak support for the inferences derived from the use of xxSSE models to our empirical data. On the other hand, if the decoupled dynamics simulations produce a scenario very different then the ones recovered in the empirical analysis the empirical heterogeneity in the trees should not affect the performance of xxSSE models and our empirical analysis can be considered robust and reliable.

To test for spurious association between diversification rates and trait states when using MuSSE models in our empirical data we thus simulated the evolution of a nine-state trait (i.e. dietary guilds) evolving in 180 randomly selected trees from the 200 trees used in the main paper (90 from each backbone resulting in equal number of trees, twenty, for each of the nine possible ancestral state). In these simulations, we used the transition rates as estimated from MuSSE rather than symmetric transition rates. The ancestral states for each of the simulations were equally distributed among the nine states (20 simulations with each state as ancestral state). From the simulations containing all guilds (n = 151; see below), we randomly sampled 40 simulations (20 from each backbone, due to computational constraints) and ran the same procedure as in the main paper to estimate speciation, extinction, and transition rates. We then counted how many simulations would show a similar dynamics to our empirical results that would have arisen from a scenario where rates of diversification were not associated with trait states.

A2) Posterior predictive simulations to test for model adequacy

For the second set of tests, we simulated 1000 trees using the *tree.musse* function from the *diversitree* package. These trees were simulated using the mode of the empirical posterior distributions of all rates. This simulation function requires an ancestral state to be given to each simulation. Hence, following Price et al. 2012 SI, we have used the proportions of all 9 diets seen on extant species as ancestral states (supplementary table 1). We then calculated the proportion of each diet in each simulated tree, and checked whether the proportion between diets created by the simulations lies around the proportion found in extant species as a way of checking for the adequacy of the estimated parameters.

A3) Model fitting in sub-clades

According to Rabosky & Goldberg (2015), if the macroevolutionary patterns we observe in a given tree are created by spurious association between trait states and diversification, the chances of observing the same macroevolutionary pattern in both the whole tree and in sub-clades of the same tree are quite low. Therefore in the last set of tests, we selected 4 of the 5 major bird orders (namely Passeriformes, Piciformes, Psittaciformes and Charadriiformes) as in Rolland et al. (2014) (28). We did not use the order Apodiformes because it is predominantly composed by either insectivorous or nectarivorous species, with very few exceptions. Hence, testing for associations between traits and diversification in Apodiformes would (a) not include the dietary guild of omnivores, and (b) require the BiSSE model (which has been shown to have serious biases and hence was not used in our analysis).

For each one of the 4 selected sub-clades we used the same 10 randomly selected trees mentioned above, and the same protocol used to analyze the whole tree. Hence, we ran each MCMC chain for 2000000 generations, sampling every 1000th iteration. The combined posterior distribution of all rates (speciation, extinction, net diversification rates) and also the posterior distribution of the differences between the rates in each present diet in relation to omnivores in each of the 4 sub-clades were then analyzed to check whether or not different diversification regimes are associated with omnivory as apparent for the whole tree.

B) Sensitivity analysis for dietary classification

To verify if our results are robust to different dietary classifications (especially concerning omnivorous species), we performed the same analysis as in our main analysis, but using a new threshold for assigning a species as an omnivore. Before running this sensitivity analysis, we investigated the effect of different dietary classifications on the average diversity of the omnivore diet, to characterize how different classification schemes would change the level of "generalization" for the omnivore diet and see which new schemes would consist on scenarios worth investigating in further detail. We used the original dataset

(scores greater than 5) and created four other datasets (main diet scores greater than 6, 7, 8 and 9), representing different degrees of restriction to the "omnivore" guild. In the five datasets we characterized the degree of diet diversity for the omnivore dietary guild by calculating the Shannon index using the scores of all food items for each omnivore species (note that the definition of omnivory and hence the number of omnivore species changes among different datasets). We then compared the distribution of Shannon indexes of omnivorous species in the five datasets to identify the best schemes to perform a sensitivity analysis. Results showed that for the first three thresholds the average dietary diversity of omnivores decreased gently (Supplementary Figure 20), but a steeper change in the average diet diversity was apparent when using scores of 8 or 9 as a threshold (highly specialized species). Given this result we focused our sensitivity analysis on the other lower categories (6 and 7). Due to computational limitations, we used only the dataset with score 7 as a threshold for the sensitivity analysis because this provided the highest sensible option. We should also note that the threshold value used in our main analysis (50%) maximizes the average diet diversity among omnivorous species (Supplementary Figure 20).

For the sensitivity analysis, we ran the MuSSE analysis for the 10 randomly selected trees using this new dietary classification. To evaluate the results we combined the posterior distributions of all rates (speciation, extinction, net diversification and transition rates) and also the posterior distribution of the differences between speciation, extinction and net diversification rates of each guild in relation to omnivores, to check for significant differences in these rates. As in the main analysis, the results for scavengers are not included due to the small number of species and the small statistical power to reliably estimate the rates. The results for the sensitivity analysis are shown in supplementary figures 20, 21, 22, 23 and 24.

C) Comparison of backbone trees

Two new high-order phylogenies were recently published aiming to resolve the deep-time phylogenetic relationships in birds (1, 2). To examine how different the two backbones (Hackett, Ericson) used by Jetz et al. (32 - both used

in our work) are to these new trees, we selected two random trees from each backbone (note that all trees within the same backbone will have the identical higher-level organization) and then pruned those trees down to a tree that contained the same species of both new trees: 1- Jarvis et al (1) with 48 terminals; 2- Prum et al (3) with 198 terminals. The trees were then used to calculate the Robinson-Foulds distance between all three pairwise combinations within each tree size class (4). This distance represents differences in topology between two trees, and is calculated by counting the number of permutation operations that need to be performed to transform one tree into the other (3). Since the difference between trees is expected to grow with tree size, these distances cannot be compared between different tree-size classes (i.e. here the trees with 48 and 198 terminals, respectively). Moreover, these Robinson-Foulds distances cannot be easily interpreted without a reference scenario. We therefore simulated 1000 random topologies for each tree size (48 and 198 terminals) using a constant-rate birth-death model, and later calculated the distances between each of the three empirical trees in each size class (Ericson, Hackett and Jarvis for the 48 terminals class, and Ericson, Hackett and Prum for the 198 terminals class), using all 1000 simulated trees from the respective size. This was done to provide a maximum expected value of distances for each size class, so that the absolute distance values could be better compared (Supplementary Figure 25).

Supplementary references

1. Davis, M. P., Midford, P. E., Maddison, W., Exploring power and parameter estimation of the BiSSE method for analyzing species diversification. *BMC Evolutionary Biology,* **13,** 38.
2. Jarvis, E. D. *et al.* Whole-genome analyses resolve early branches in the tree of life of modern birds. *Science (80-.).* **346,** 1320–1331 (2014).
3. Prum, R. O. *et al.* A comprehensive phylogeny of birds (Aves) using targeted next-generation DNA sequencing. *Nature* **526,** 569–573 (2015).
4. Robinson, D. F. & Foulds, L. R. Comparison of phylogenetic trees. *Math. Biosci.* **53,** 131–147 (1981).
